# regSNPs-splicing: a tool for prioritizing synonymous single-nucleotide substitution

**DOI:** 10.1007/s00439-017-1783-x

**Published:** 2017-04-08

**Authors:** Xinjun Zhang, Meng Li, Hai Lin, Xi Rao, Weixing Feng, Yuedong Yang, Matthew Mort, David N. Cooper, Yue Wang, Yadong Wang, Clark Wells, Yaoqi Zhou, Yunlong Liu

**Affiliations:** 1 School of Informatics and Computing, Indiana University, Bloomington, IN 47408 USA; 2 Center for Computational Biology and Bioinformatics, Indiana University School of Medicine, 410 w 10th st, HS 5000, Indianapolis, IN 46202 USA; 3 Pattern Recognition and Intelligent System Institute, Automation College, Harbin Engineering University, Harbin, 150001 Heilongjiang China; 4 School of Informatics and Computing, Indiana University Purdue University Indianapolis, Indianapolis, IN 46202 USA; 5 School of Information and Communication Technology, Gold Coast Campus, Griffith University, Southport, QLD 4222 Australia; 6 Institute of Medical Genetics, Cardiff University, Heath Park, Cardiff, CF14 4XN UK; 7 Departments of Medical and Molecular Genetics, Indiana University School of Medicine, Indianapolis, IN 46202 USA; 8 School of Computer Science and Technology, Harbin Institute of Technology, Harbin, 150001 Heilongjiang China; 9 Department of Biochemistry and Molecular Biology, Indiana University School of Medicine, Indianapolis, IN 46202 USA; 10 Institute for Glycomics and School of Informatics and Communication Technology, Griffith University, Parklands Dr., Southport, QLD 4215 Australia; 11 Center for Medical Genomics, Indiana University School of Medicine, Indianapolis, IN 46202 USA

## Abstract

While synonymous single-nucleotide variants (sSNVs) have largely been unstudied, since they do not alter protein sequence, mounting evidence suggests that they may affect RNA conformation, splicing, and the stability of nascent-mRNAs to promote various diseases. Accurately prioritizing deleterious sSNVs from a pool of neutral ones can significantly improve our ability of selecting functional genetic variants identified from various genome-sequencing projects, and, therefore, advance our understanding of disease etiology. In this study, we develop a computational algorithm to prioritize sSNVs based on their impact on mRNA splicing and protein function. In addition to genomic features that potentially affect splicing regulation, our proposed algorithm also includes dozens structural features that characterize the functions of alternatively spliced exons on protein function. Our systematical evaluation on thousands of sSNVs suggests that several structural features, including intrinsic disorder protein scores, solvent accessible surface areas, protein secondary structures, and known and predicted protein family domains, show significant differences between disease-causing and neutral sSNVs. Our result suggests that the protein structure features offer an added dimension of information while distinguishing disease-causing and neutral synonymous variants. The inclusion of structural features increases the predictive accuracy for functional sSNV prioritization.

**Electronic supplementary material:**

The online version of this article (doi:10.1007/s00439-017-1783-x) contains supplementary material, which is available to authorized users.

## Introduction

While single-nucleotide variants (SNVs) underlie a myriad of diseases, synonymous SNVs (sSNVs) that do not alter which amino acid is encoded have traditionally been assumed to have little or no biological impact. However, recent work suggests that sSNVs may contribute to disease pathogenesis by affecting the affinity of RNA-binding proteins to disrupt RNA processing and/or translational control (Wan et al. 2014). The importance of synonymous point mutations in cancer has been further demonstrated by a recent survey based on roughly 4000 cancer exomes from 19 cancer types, which showed a significant enrichment of synonymous mutations in oncogenes, as compared to non-cancer genes with matched genomic features (Supek et al. 2014; Li et al. 2016; Xiong et al. 2015; Cartegni et al. 2002; Sauna and Kimchi-Sarfaty 2011; Duan et al. 2003; Macaya et al. 2009; Chamary and Hurst 2005).

Current bioinformatics tools in prioritizing deleterious sSNVs mainly focus on the potential impacts of individual variants on splicing outcome. Such methods often derive a series of genomic features describing how a candidate variant can potentially affect splicing regulation, and attempt to use these features to predict either disease relevance, or splicing outcome, as measured by large-scale RNA-seq experiments. Despite the positive prediction power in prioritizing disease-causing sSNVs, such methods, however, do not consider whether the affected splicing events will result in major protein function changes (Mort et al. 2014). As demonstrated in our previous analysis on non-frame shifting micro-insertions/deletions (INDELs), inclusion or exclusion of a stretch amino-acid sequences does not guarantee the functional changes of affected protein, unless they occur within key structural elements of the protein (Zhao et al. 2013). In addition, recent surveys also suggest that many splicing variations are crucial to the protein functions and organismal phenotypes (Xiong et al. 2015; Kelemen et al. 2013; Rivas et al. 2015; Zheng and Black 2013; Faustino and Cooper 2003).

In this study, we hypothesize that considering the exon-specific protein structure features will significantly increase the accuracy of the prediction. Using potential disease-causing and neutral data sets derived from the human gene mutation database (HGMD), ClinVar, and 1000 Genomes projects, we systematically evaluated hundreds of genomics and protein structure features that are associated to individual synonymous SNVs. Our results suggest that including protein structure features dramatically increases our ability for identifying disease-causing synonymous SNVs.

## Results

### Training data set

We constructed a training data set that includes both disease-causing and neutral sSNVs. The disease-causing sSNVs were selected from the human gene mutation database (HGMD) (Stenson et al. 2014), and the neutral sSNVs were selected from the 1000 Genomes database (Genomes Project C et al. 2012). As of September 2014, the HGMD database contains 1111 deleterious synonymous mutations that affect splicing, of which 697 locate on the splice sites (+1/+2/+3 loci in donor site and −1 locus in acceptor site), and 414 reside inside the exon but off the splice sites. These two types of sSNVs are referred as variants on splice site consensus (VSS) and variants in internal exons (VIE), respectively.

Most of the variants in our training data set are in the DM (disease-causing) category with direct evidence of being disease-causing mutations. Specifically, out of 697 VSS HGMD variants, 656 (94.1%) are in the DM (disease-causing) category, and out of 414 VIE HGMD variants, 344 (83.1%) are from DM (disease-causing) category. The overall distribution of categories is shown in Fig. S1. Since these two types of variants may affect splicing regulation with different mechanisms, with VSS variants more likely to directly interfere with the formation of the splicesome, while VIE variants playing more roles in affecting RNA-binding protein (RBP) binding, their impacts on splicing regulation were evaluated separately. To avoid inflating the over-representation of certain genomic features due to the occurrences of multiple variants in the same affected exon, we randomly select only one variant per exon in the further analysis. This process results in a total of 980 deleterious sSNVs in the HGMD database, of which 651 and 329 locate on and off splice sites, respectively. Similar as our earlier study on INDELs (Zhang et al. 2014), the neutral sSNVs were selected from the 1000 Genomes Project, in which genotyped individuals did not exhibit any apparent disease phenotypes. The 1000 Genomes data contain 2582 VSS and 66,900 VIE variants, respectively. To minimize false positives in the neutral group of the training set, we only selected those sSNVs with a minor allele frequency (MAF) greater than a threshold (3% for VSS and 10% for VIE variants). The overall gold standard data set includes 651 disease-causing and 399 neutral VSS variants, and 329 disease-causing and 7231 neutral VIE variants, respectively. To make a balanced training set, we randomly selected the same number of negative data set as positive data set to train and test our models. To evaluate features that are associated with disease-causing and neutral sSNVs, and build computational model for novel variant prioritization, we used 2/3 of our data set as training data, and the remaining 1/3 as independent test data.

### Disease-causing variants tend to impact splicing regulation

We evaluated a broad array of features that can be classified into three major categories: genomic features characterizing how individual sSNVs affect splicing regulation, the structural features evaluating how the inclusion/exclusion of alternatively spliced exons affect protein function, and others (such as conservation). A detailed list of features and how they are derived can be found in the supplementary materials and online methods. As reported in the previous studies, features characterizing how sSNVs affect splicing regulation play important roles in distinguishing disease-causing and neutral variants (Barash et al. 2010). For instance, among the 201 RNA-binding proteins (RBPs) with known position weight matrices (PWMs) (Ray et al. 2013), disease-causing sSNVs showed greater alteration on RBP binding in terms of specific diseases, comparing with neutral variants (Fig. S2). This is consistent on both the magnitude of matching score changes, and the probability that an sSNV changes RBP binding (detailed calculation methods can be found in online methods, Fig. S3). Similarly, other features associated with individual variants, such as the RNA secondary structure features on the variant loci, the inherent strength of 5′- and 3′-splicing sites of exons containing candidate variant, the distance between the variant loci and splicing junction, and the ability of the variants disrupting the cluster of exonic splicing enhancers and silencers, all have statistically significant prediction power for distinguishing disease-causing and neutral variants, as evaluated by the Matthew’s correlation coefficient (MCC), and Kolmogorov–Smirnov (K–S) test (Supplement Table 1; Fig. S4).

### Disease-causing variants tend to locate within the exons of key structural regions

**Fig. 1:**
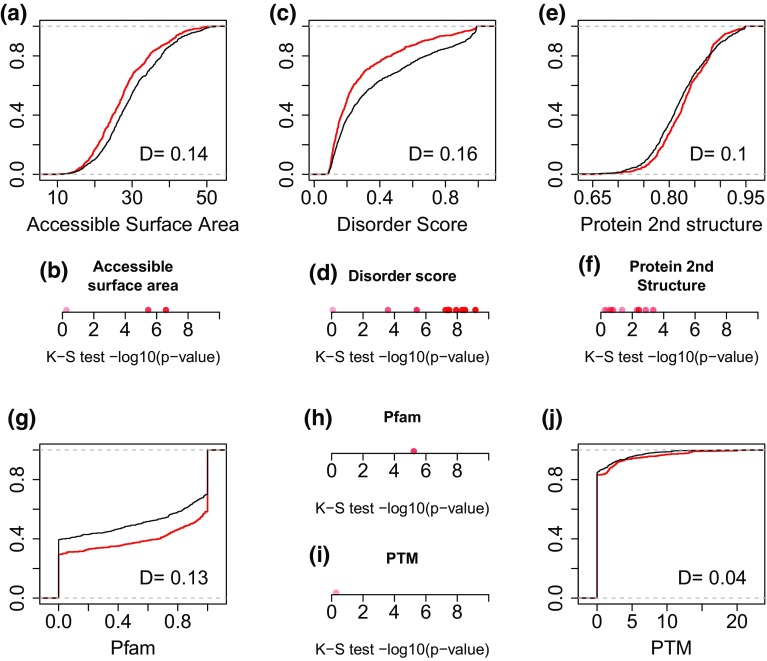
Cumulative probability density function (CDF) curves and Kolmogorov–Smirnov (K–S) test *p* values on various protein structure features for the exons containing disease-causing (*red*) and neutral (*black*) sSNVs. **a** CDF of the average solvent accessible surface area (ASA) of all the amino-acid residuals in the exon. **b** KS–S test *p* values for the average, minimum and maximum ASA values of all the amino-acid residuals in the exon. **c** CDF of the average disorder score of all the residuals in the affected exon. **d** K–S test *p* values for 12 disorder score-derived features (Supplementary Table 1). **e** CDF of the average probability of the most likely protein secondary structure (alpha-helix, beta sheet, or random coil) on all the residuals in the affected exon. **f** K–S test *p* value for 12 protein secondary structure-derived features (Supplementary Table 1). **g**, **h** CDF and K–S *p* values of the percentage of the exon overlapping with known/predicted Pfam domain. **i**, **j** CDF and K–S *p* values of the normalized PTM counts in the affected exon

In addition to the genomic features related to splicing regulation, we have observed strong prediction power for the measurements characterizing the protein structure features of the exons containing the putative variants. Disease-causing sSNVs tend to locate in the exons with lower average solvent accessible surface areas (ASA), indicating that they are more likely to be in buried core protein regions (K–S test *p* value = 2.6 × 10^−7^, Fig. 1a, b, Fig. S5). In addition, comparing to neutral variants, disease-causing ones are also under-represented in the exons in intrinsic disorder regions (K–S test *p* value = 7.0 × 10^−10^, Fig. 1c, d, Fig. S6), suggesting that they are more likely to be in the structural regions. Consistent with these observations, disease-causing variants tend to reside in the exons with higher percentage of overlapping with known or predicted protein family domains (K–S test *p* value = 5.9 × 10^−6^, Fig. 1g, h, Fig. S7). As for the protein secondary structures, exons containing disease-causing sSNVs are enriched for alpha-helix (K–S test *p* value = 0.004), and random coil (K–S test, *p* value = 0.001) (Fig. 1e, f, Fig. S8). All these observations strongly suggest that, in addition to features related to splicing regulation, protein structure features on the variant-containing exons can provide additional layer of information in distinguishing disease-causing and neutral variants.

### Prioritizing sSNVs based on their impact of splicing regulation and protein structure

**Fig. 2:**
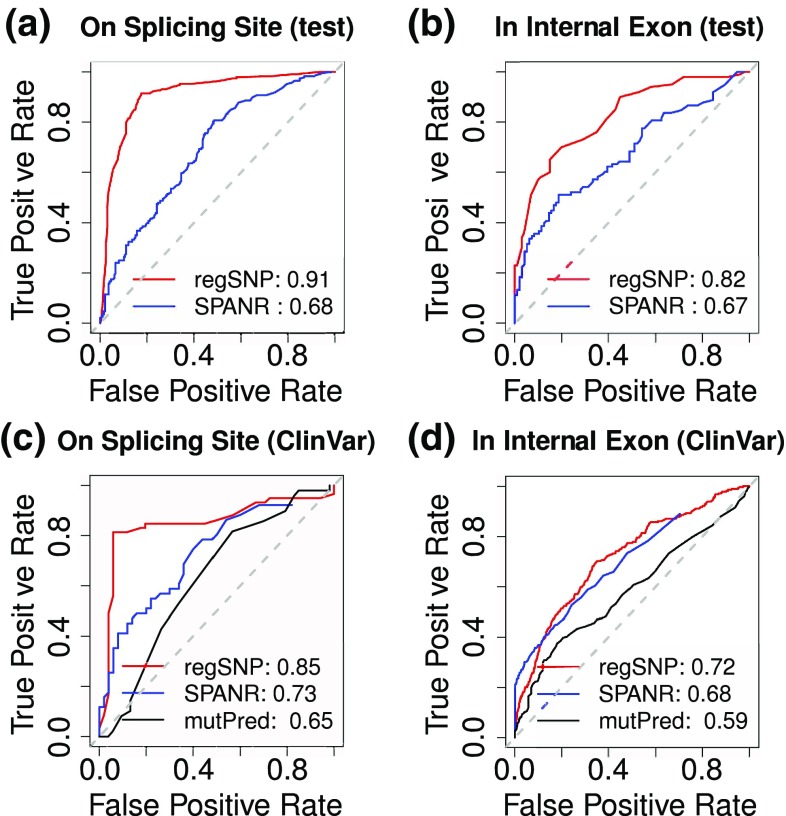
Comparison between regSNP-splicing and SPANR on independent test variant data set and ClinVar variant data set. **a**, **b** ROC curves showing the performance of regSNP-splicing (*red curve*) and SPARN (*blue curve*) on an independent test data sets for VSS, and VIE variants, respectively. **c**, **d** ROC curves showing the performance of regSNP-splicing (*red curve*), SPARN (*blue curve*) and mutPred Splice (*black curve*) for VSS and VIE variants documented in the ClinVar database, respectively

Based on the aforementioned evaluation, a random forest algorithm was employed for building a prediction model for distinguishing disease-causing and neutral sSNVs. We evaluated the model prediction using an independent test data set that is not used in model training. The test data sets for on- and off-splicing site sSNVs include 232 and 100 pairs of disease-causing and neutral sSNVs, respectively. For VSS variants (on-splicing site variants), the MCC and AUC values using the whole feature set were 0.67 and 0.91, respectively. For VIE variants (off-splicing site variants), the MCC and AUC values were 0.47 and 0.82, respectively. To compare our algorithm with available tools focusing only on the effects of *sSNVs* on splicing outcome, but not on the structural features of alternatively spliced exons, we applied SPANR (Splicing-based Analysis of Variants), a tool for evaluating how SNVs cause splicing mis-regulation, on our independent test data set (Xiong et al. 2015). We also compared with a previously published tool mutPred Splice (Mort et al. 2014). In our study, we used the maximum mutation-induced change in PSI across 16 tissues which is reported by SPANR by default. In addition, we used the general score reported by mutPred as an indicator of disease-causing probability. In both cases (on- and off-splicing sites), our algorithm significantly out-performed SPANR and mutPred Splice in distinguishing disease-causing and neutral variants (Fig. 2). The areas under curve (AUCs) for regSNPs-splicing and SPANR are 0.91 and 0.68 for VSS variants, and 0.82 and 0.67 for VIE variants, respectively. In addition, mutPred Splice has AUC as 0.65 for VSS variants and 0.59 for VIE variants. We have also tested our algorithm on the pathogenic and benign synonymous SNVs documented in the ClinVar database. For VSS variants, similar to the test in our independent test data set, regSNPs-splicing demonstrated significantly improved performance than SPANR; AUCs for these two algorithms are 0.85 and 0.73, respectively. For the variants that are not on the splice site (VIE variants), however, the performances of the two algorithms are similar (AUCs for regSNPs and SPANR are 0.70 and 0.68, respectively). One possible explanation for this is that most benign sSNVs in the ClinVar database do not change splicing outcome; based on SPANR prediction, among 3703 benign sSNVs, only 3 (0.08%) can cause more than 20% change of splicing inclusion (|dPSI| ≥0.2). Based on the rationale of the model design, regSNPs-splicing works more effective if the pathogenic variants contain substantial amount of variants that do cause splicing change, while the resultant splicing change does not cause protein function changes.

### Minor allele frequency is reversely correlated with disease-causing probability

**Fig. 3:**
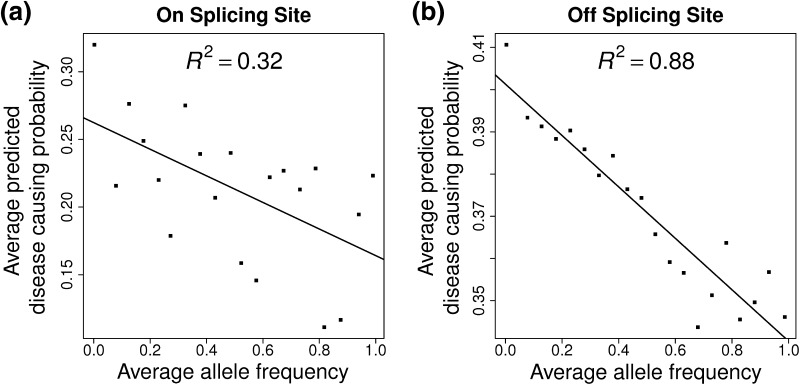
Reverse correlation between average minor allele frequency (MAF) and average predicted disease-causing probability for **a** on-splicing site and **b** off-splicing site 1000 Genomes variants, respectively. Minor allele frequency, ranging between 0 and 1, is divided into 20 equal bins, and each bin represents 0.05 increment of MAF. For all the variants with MAF falling into each bin, we calculated their average MAF and average disease-causing probability values. *One dot* represents a pair of average MAF and average DCP. A linear model was fitted for the 20 dots and *R* ^2^ value is calculated

We obtained allele frequencies for all the synonymous variants from the 1000 Genomes Project data. The allele frequency in the population should, in general, reflect the occurrences of that allele with respect to its putative biological function. As expected, there was a strong negative correlation between the predicted disease-causing probability and allele frequency for both VSS and VIE variants with correlation coefficient = 0.32 and 0.88, respectively (Fig. 3a, b).

### Web-based analysis portal

We provide a Web access to our tool (http://watson.compbio.iupui.edu/regSNP-splicing/) and also a download link of the source code and database annotation files.

## Discussion

The genetic code error arising from a single silent nucleotide variation can be populated through transcriptional regulation, post-transcriptional regulation, and translation process. The mis-splicing of exon in mRNA eventually will change protein structure and affect protein’s function. Many disease phenotypes can be traced back to that “nucleotide switch” which triggered dramatic alteration of biological processes (Milenkovic et al. 2010; Akiyama et al. 2007; Neveling et al. 2012; Banerjee et al. 2011; Leontiou et al. 2008). To elucidate the subsequent abnormal biological process which are implemented with aberrant genetic information, several studies have investigated the genetic context of nucleotide sequence around sSNV and further extend to a comprehensive profile of affected splicing regulation elements (Kurmangaliyev and Gelfand 2008; Ward and Cooper 2010; Baralle and Baralle 2005). Despite of these efforts, the examination of protein function and structural integrity is largely dismissed yet providing a direct interpretation of disease mechanism. In addition to the genomic sequence including length of exon and intron, proximity to exon boundary, conservation, exon junction site strength, and splicing regulation motifs, we extend our scope to protein structure and function features: solvent accessible surface area, protein secondary structure, intrinsic disorder score, Pfam domains, and post-translation modification sites. Our analyses confirmed the conclusions from the previous studies (Scott et al. 2012; Ward and Kellis 2012; Pagani et al. 2005; Woolfe et al. 2010) that synonymous SNVs can have great influence on the splicing regulation. We have observed significant differences on a broad array of genomic features that are associated with disease-causing and neutral sSNVs, respectively. Importantly, our results strongly suggest that the protein structure features offer an added dimension of information while distinguishing disease-causing and neutral synonymous variants. The inclusion of structural features increases the predictive accuracy for functional *sSNV* prioritization.

In our research, we specifically split our data set into two parts: one set of sSNVs defined as “on consensus splicing site” and it contains sSNVs which are very close (within 3 nucleotides to donor site or one nucleotide to acceptor site). In addition, the other set of sSNVs are defined as “variants in internal exon” which contains sSNVs that are >3 nucleotides from donor site and >1 nucleotide from acceptor site. For the data set “on consensus splicing site”, we did not use the proximity to exon boundary as a feature for model training, since it is a very strong indicator to distinguish whether a synonymous mutation is disease-causing or neutral. Therefore, our model for the variant on splicing site does not learn the position of sSNVs. For the data set “in internal exon”, we examined the proximity from the location of sSNV to the closer splicing junctions and we can tell that proximity is a useful feature but not overwhelming (plot h, Fig. S4). Therefore, for the data set “in interval exon”, our model does not just learn the position of sSNV either.

To evaluate the performance of our tool in predicting disease-causing mutations, we have compared our tool with SPANR and mutPred Splice (Mort et al. 2014). SPANR and mutPred Splice were primarily designed to quantify splicing level change of one exon in the presence of single-nucleotide variation. Although SPANR and mutPred Splice do not have a specific disease focus, the splicing level change is a strong indicator of disease relevance (Ward and Cooper 2010). Therefore, both tools are capable of predicting disease-causing mutations based on same rationale.

As a direct evaluation of the importance of protein feature, we evaluated the power of protein level information separately. We divided all the features into three categories: DNA evolution, splicing regulation features, and protein function and structure features. For every category, we trained and tested one single model based on tenfold cross validation. For the model built using nucleotide evolution, we plotted ROC curve, and the AUC is 0.56 for VSS variants and 0.59 for VIE variants. For the model built using splicing regulation features, the AUC is 0.91 for VSS variants and 0.81 for VIE variants. For the model built using protein features, the AUC is 0.67 for VSS variants and 0.71 for VIE variants. Therefore, for both VIE and VSS variants, protein function and structure features have been demonstrated of strong classification power (Fig. S9).

To make our model independent of gene, we have strictly controlled sequence similarity in our model training and testing process. In the original HGMD and 1000 Genomes Project data set, there are scenarios that multiple variants originate from the same exon. To avoid over-representation of such exons and genes in our models, we kept only one variant per exon in both training and testing data. Furthermore, we performed a more strict control of sequence similarity in our training and testing data set. For each gene family, we only selected the gene with most number of exons and then we only keep variants in this gene. And then, we trained our models using the remaining data. Using tenfold cross-validation strategy, the model for VSS has achieved AUC as 0.93 and the model for VIE has AUC as 0.84 (Fig. S10). We demonstrated that our models were not significantly affected by sequence similarity.

In our current model, the structural information on the potential disease-causing isoform is not calculated. The first reason is that we believe that evaluating the structural information on the naturally occurring splicing events has provided enough sensitivity to our approach. In addition, more importantly, for practical purpose, calculation for structural information based on amino-acid sequences is very time-consuming. To make the tool usable to general public, most of the protein features are pre-calculate based on the current gene annotation.

However, our models also have some limitations. One of the limitations is that our training data size of HGMD is not large enough. This can be improved with the growth of the databases of HGMD and ClinVar in the future. Another limitation is that the protein structure features are all prediction based. This is reasonable, otherwise using known protein structures information in PDB database will limit the training data set size and impose difficulty on implementation of both model training and testing. However, this would add another level of inaccuracy.

## Methods

### Training data sets

A “gold standard” data set for the development of machine learning-based prediction algorithms was obtained from the human gene mutation database (HGMD) and the 1000 Genomes Project. The positive training set was acquired from the HGMD database, which contains 1373 disease-causing synonymous SNVs (sSNVs) that have experimentally been verified to cause disease through affecting the processes of alternative splicing. We further removed the variants reside in the first exon and last exon of a gene, whose inclusion/exclusion status is often regulated through mechanisms other than splicing regulation, such as alternative promoter, or alternative polyadenylation. The positive and negative data sets may be biased by repetitively appeared genes and exons which can introduce highly homologous sequences into our training data sets. To avoid the over-representation of certain exons due to the occurrences of multiple disease-causing variants, we only keep one variant per exon. Therefore, one exon is used only once for training and testing purposes. The remaining 980 disease-causing sSNVs were further classified into two groups, the one locating at +1/+2/+3 nucleotides on the donor side, and −1 nucleotide on the acceptor side is considered as on consensus splice site variants (VSS), while the other variants are considered as in internal exon (VIE). We have also removed the variants appearing in the ClinVar database for training purpose. This classification resulted in 651 VSS and 329 VIE disease-causing sSNVs, respectively.

The negative training set, i.e., “neutral” synonymous variants, was acquired from the 1000 Genomes Project, in which genotyped individuals did not exhibit any apparent disease phenotypes. To minimize false positives, we selected only those sSNVs with a minor allele frequency (MAF) greater than 10% for VIE variants, and 3% for VSS variants. The reduced MAF cutoff for VSS variants is implemented due to the limited available number of on splicing site sSNVs in the 1000 Genomes database. This selection criterion resulted in 7231 neutral VIE variants, and 329 VSS variants, respectively.

In addition to the HGMD database, the synonymous variants documented in the ClinVar database were used as test data set for model evaluation. The current ClinVar database contains 4765 synonymous variants, of which 230 and 4535 are pathogenic and benign, respectively. To avoid potential evaluation bias due to the overlapping records between HGMD and ClinVar, we have removed the overlapping variants from the training data set. Although the total number of usable sSNVs in ClinVar database is limited, it offers the opportunity to validate the prediction accuracy from an independent test data set.

### Feature description

We evaluated a broad array of features that can be classified into three major categories: genomic features characterizing how individual SNVs affect splicing regulation, structural features evaluating how the inclusion/exclusion of alternatively spliced exons affect protein function, and others (such as conservation). A detailed list of features can be found in Supplementary Table 1.

#### Genomic features

##### Potential impacts of sSNVs on the binding affinities of RNA-binding proteins (RBPs)

For a given sSNV, its effect on the binding of a particular RBP (RNA-binding protein) will be evaluated by the differences in the RBP-binding scores between reference and alternative alleles. The RBP-binding score was calculated based on the RNA sequence and the RBP position weight matrix (PWM) documented in the RBPDB and cisBP-RNA databases; collectively, these two databases contain the PSSMs of 201 RNA-binding proteins (Ray et al. 2013; Cook et al. 2011). A PWM is a matrix of values that gives the count of each nucleotide at each locus of the binding site. The binding affinity between the n-nt RNA sequence, and the PWM is described by a matching score S as follows:

1 $$S = \sum\limits_{i = 1}^{k} {\sum\limits_{{j \in \{ A,T,G,C\} }} {\log_{2} \frac{{(n_{ij} + c_{ij} )/(N + \sum\nolimits_{j = 1}^{4} {c_{ij} } )}}{{d_{j} }}} } ,$$

where *n*_*i,j*_ is the count of the *j*th nucleotide on the *i*th position in one PWM, *c*_*i,j*_ is the pseudocount for the *j*th nucleotide on the *i*th position in the PWM, and *d*_*j*_ is the prior base frequency for the *j*th nucleotide (*d*_*j*_ = 0.25 for *j* = A, T, G, C). *N* is the total number of experimentally validated binding sites for one RBP, and *k* is the width of the binding site.

In Eq. (1), a high or low matching score indicates that the putative sequence has, respectively, a high or low likelihood to be a potential binding site. Each position of a binding site is assumed to be independent of the other. The matching score distributions for binding and non-binding events were both estimated based on PSSM of an individual RBP. We assume that the matching score follows a Gaussian distribution, with mean as *M*_s_ and variance as *V*_s_. The mean and variance of the binding scores for specific RBP-binding events are defined as follows:

2 $$s_{ij} = \log_{2} \frac{{(n_{ij} + c_{ij} )/(N + \sum\nolimits_{j = 1}^{4} {c_{ij} } )}}{{d_{j} }},$$

3 $$M_{\text{s}} = \sum\limits_{i = 1}^{k} {\sum\limits_{j \in (A,T,G,C)} {f_{ij} \times s_{ij} } } ,$$

4 $$V_{\text{s}} = \sum\limits_{i = 1}^{k} {\sum\limits_{{j \in \{ A,T,G,C\} }} {f_{ij} \times s_{ij}^{2} - (f_{ij} \times s_{ij} )^{2} } } .$$

In Eq. (2), the score *s*_*i,j*_ is the value of the *i*th column and the *j*th row of the position specific score matrix (PSSM), which is defined as the logarithmic ratio of the percentage of the *j*th nucleotide (A, C, G, or U) in column *i* of the binding sites to the percentage in random sequence. In this equation, *n*_*i,j*_ is the count of the *j*th nucleotide on the *i*th position in the PWM, *c*_*i,j*_ is the pseudocount for the *j*th nucleotide on the *i*th position in the PWM. *N* is the total number of experimentally validated binding sites for each RBP. *d*_*j*_ is the prior base frequency for the *j*th nucleotide (*d*_*j*_ = 0.25 for *j* = A, T, G, C).

In Eqs. (3) and (4), *f*_*i,j*_ is the approximation of the true frequency of each nucleotide at each binding locus. For binding events,

5 $$f_{ij} = \frac{{2^{{s_{ij} }} }}{4},$$

and for non-binding events, $f_{i,j }$ = 0.25.

As defined in our previous study on transcription factors and micro-INDELs, the magnitude (*M*) of a sSNV affecting the binding of an RBP is defined as a likelihood ratio of the sSNV affected loci being a binding event as opposed to it being a non-binding event in reference and alternative forms, respectively:

6 $$\begin{aligned} M = { \log }_{2} \frac{{P\left( {S_{A} |B} \right)/P(S_{A} |{\text{NB}})}}{{P\left( {S_{R} |B} \right)/P(S_{R} |{\text{NB}})}} \hfill \\ = { \log }_{2} \left( {\frac{{\mathop \smallint \nolimits_{ - \infty }^{{S_{A} }} \frac{1}{{\sqrt {2\pi V_{\text{S}} } }}e^{{ - \frac{1}{2}\left( {\frac{{x - M_{\text{S}} }}{{V_{\text{S}} }}} \right)^{2} }} d(x)/\left( {1 - \mathop \smallint \nolimits_{ - \infty }^{{S_{A} }} \frac{1}{{\sqrt {2\pi V'_{\text{S}} } }}e^{{ - \frac{1}{2}\left( {\frac{{x - M'_{\text{S}} }}{{V'_{\text{S}} }}} \right)^{2} }} d(x)} \right)}}{{\mathop \smallint \nolimits_{ - \infty }^{{S_{R} }} \frac{1}{{\sqrt {2\pi V_{\text{S}} } }}e^{{ - \frac{1}{2}\left( {\frac{{x - M_{\text{S}} }}{{V_{\text{S}} }}} \right)^{2} }} d(x)/\left( {1 - \mathop \smallint \nolimits_{ - \infty }^{{S_{R} }} \frac{1}{{\sqrt {2\pi V'_{\text{S}} } }}e^{{ - \frac{1}{2}\left( {\frac{{x - M'_{\text{S}} }}{{V'_{\text{S}} }}} \right)^{2} }} d(x)} \right)}}} \right) \hfill \\ \end{aligned}$$

where *R* and *A* indicates the reference and mutated sites, respectively; *B* and NB denote binding and non-binding events, respectively. *S*_R_ and *S*_A_ each represents the matching scores of the reference and mutated sites. $P\left( {S_{A} |B} \right)$ is the probability of matching score $S_{A}$ of mutated site when it is a binding event, and $P\left( {S_{A} | {\text{NB}}} \right)$ is the probability of $S_{A}$ when it is a non-binding event. Similarly, $P\left( {S_{R} |B} \right)$ is the probability of matching score $S_{R}$ of reference site when it is a binding event and $P\left( {S_{R} | {\text{NB}}} \right)$ is the probability of matching score $S_{R}$ for non-binding event. $M_{\text{S}}$ and $V_{\text{S}}$ are, respectively, the mean and variance of the matching score for binding events, and $M'_{\text{S}}$ and $V'_{\text{S}}$ are the mean and variance of the matching score of non-binding events. A positive *M* score indicates a gain of an RBP-binding site, whereas a negative *M* score indicates the loss of an RBP-binding site.

We further calculate a Bayesian-based posterior probability for RBP-binding-site gain/loss, defined as the probability that a genetic locus could switch between binding and non-binding status, with and without the synonymous variant:

7 $$\begin{aligned} P = P\left( {R = B,A = NB |S_{R} , S_{A} } \right) + P\left( {R = NB,A = B |S_{R} , S_{A} } \right) \hfill \\ = \frac{{P\left( {R = B,A = NB} \right)P\left( {S_{R} ,S_{A} |R = B,A = NB} \right)}}{{P\left( {S_{R} , S_{A} } \right)}} + \frac{{P\left( {R = NB,A = B} \right)P\left( {S_{R} ,S_{A} |R = NB,A = B} \right)}}{{P\left( {S_{R} , S_{A} } \right)}} \hfill \\ = \frac{{P\left( {R = B} \right)P\left( {A = NB} \right)P\left( {S_{R} |R = B} \right)P\left( {S_{A} |A = NB} \right)}}{{P\left( {S_{R} } \right)P\left( {S_{A} } \right)}} + \frac{{P\left( {R = NB} \right)P\left( {A = B} \right)P\left( {S_{R} |R = NB} \right)P\left( {S_{A} |A = B} \right)}}{{P\left( {S_{R} } \right)P\left( {S_{A} } \right)}} \hfill \\ = \frac{{P\left( B \right)\left( {1 - P\left( B \right)} \right)[P\left( {S_{R} |R = B} \right)P\left( {S_{A} |A = NB} \right) + P\left( {S_{R} |R = NB} \right)P\left( {S_{A} |A = B} \right)]}}{{P\left( {S_{R} } \right)P(S_{A} )}} \hfill \\ = \mathop \smallint \limits_{0}^{1} [P\left( B \right)\left( {1 - P\left( B \right)} \right)(P\left( {S_{R} |R = B} \right)P\left( {S_{A} |A = NB} \right) + P\left( {S_{R} |R = NB} \right)P\left( {S_{A} |A = B} \right))/P\left( {S_{R} } \right)P\left( {S_{A} } \right)d(B) \hfill \\ \end{aligned}$$

where *R* and *A* indicates whether a genetic locus is in reference or alternative form, *B* means “binding event” and NB means “non-binding event”. Therefore, *R* = *B* means that the genetic locus in its reference status is a binding site of RBP, and vice versa. Random variables *B* and NB are both assumed to follow beta distribution. We assume that *R* and *A* are independent of each other and *S*_*R*_ and S_A_ are also independent of each other. *P*(*B*) is the prior probability that a genomic region is a binding site for a RNA-binding protein. We also assume that the distribution of random variable *B* has mode value as 0.05. $S_{A}$ denotes the matching score for alternative form and $S_{R}$ denotes the matching score for reference form. We integrated over B to get the overall probability that one sSNV has changed the status of a genomic region from binding status to non-binding status, or from non-binding status to binding status.

##### RNA secondary structure features on the variant loci

RNA-binding protein binding has well-established preference on specific RNA secondary structures; some proteins tends to bind on single-stranded regions, others double-stranded regions. Such preference may provide additional specificity for RBP binding. In addition, single-nucleotide changes may disrupt the overall RNA secondary structure on the RBP-binding sites, and further affect RBP-binding affinity. For a specific sSNV, we calculated the average single-strandness probability for the nucleotides upstream and downstream 7 bases of variant locus (putative-binding sites), which is calculated using RNAfold (Lorenz et al. 2011). The changes on the RNA secondary structure caused by sSNV on the putative binding sites are calculated using RNAdistance (Lorenz et al. 2011).

##### Inherent strength of 5′- and 3′-splicing sites of exons containing candidate sSNVs

We previously reported that the sSNVs residing in the AS exons are more likely to have phenotypic consequences (Teng et al. 2011). We therefore evaluated the inherent strength of 5′- and 3′-splicing sites of exons containing candidate sSNVs. This measurement may serve as an important feature for quantifying whether the candidate splicing events require additional assistance from other RNA-binding proteins; more assistance from RBPs may be needed for an exon with weaker junction strength. The inherent splicing strength of 5′- and 3′-splicing sites are calculated based on the position weight matrices (PWMs) describing the sequence features on/around canonical splicing sites (Itoh et al. 2004).

##### ESE/ESS cluster scores

Exonic splicing motifs which consist of 6 nucleotides within an exon are categorized as exon spicing enhancer (ESE) or exon splicing silencer (ESS) based on whether they promote or prohibit splicing process, respectively. We scanned the affected exons and search for occurrences of known or predicted ESE and ESS motifs. For this purpose, we have collected 76 known motifs, 2298 predicted ESE motifs, and 1195 predicted ESS motifs (Barash et al. 2010; Fairbrother et al. 2002; Zhang and Chasin 2004). Overlapping motifs are combined and further defined as a ‘motif set’. Multiple motif sets which are located within 6 bp apart are defined as a ‘motif cluster’. Within a motif cluster, a gap ≤3 bp is denoted as a short interval—and a gap larger than 3 bp and less than or equal to 6 bp is denoted as a long interval. The total number of occurrence of short intervals is denoted as $I_{\text{s}}$ and the total count of long intervals is denoted as $I_{\text{l}}$. Then, a motif set is scored as $S_{\text{set}} = 2^{{N_{\text{m}} - 1}}$, where $N_{\text{m}}$ is the number of overlapping motifs. A motif cluster is scored based on $I_{\text{s}}$, $I_{\text{l}}$ and number of motif sets within a motif cluster, where $S_{\text{cluster}} = \left( {2 \cdot I_{\text{s}} + I_{\text{l}} + \# {\text{motif set}}} \right) + S_{\text{set}}$. $S_{\text{set}}$ is defined to measure the local density of splicing motifs within an exon, and $S_{\text{cluster}}$ is measuring the aggregation of motif sets. Finally, the enrichment of ESE and ESS within an exon using a ‘cluster score’ is defined as follows:

8 $${\text{Cluster score }} = \frac{{{ \log }^{{S_{\text{cluster}} }} }}{{{\text{exon}}\; {\text{length}}}}$$

where exon length’s unit is per 100 base pairs. The effect of a specific SNV on ESE/ESS clustering is evaluated based on the differences of cluster scores for the reference and alternative alleles, respectively.

##### Proximity to the 5′- and 3′-splicing junction

The proximity is defined as the distance from a variant and exon boundaries. Here, we separate our SNVs into two different categories: on splicing site (within 3 bp of donor sites or 1 bp of acceptor site) and off-splicing site (the other regions of exon). SNVs on splicing site mainly interferes with various molecular and affect formation of spliceosome, and the off-splicing site SNVs mainly affect the binding of splicing regulators. This feature is not used for on-splicing site variants.

#### Protein structure/function features

Disruption of protein secondary or tertiary structures is one possible reason for deleterious alternative splicing events. We, therefore, evaluated several features describing the effects of affected splicing pattern on protein structures. Such features include protein structure/intrinsic disorder scores, solvent accessible surface areas (ASA), protein secondary structures, and known and predicted protein family domains (pfam). In addition, the known post-translational modification status on the affected splicing event is also evaluated.

##### Intrinsic disordered regions

Intrinsically disordered regions are defined as a stretch of amino-acid sequences that lack the ordered tertiary and/or secondary structures. We have previously reported applying disorder score of affected protein regions in distinguishing disease-causing and neutral micro-insertion and -deletions (Zhang et al. 2014). Similarly, we measured the disorder property of affected protein regions that result from the mis-spliced exon in transcript. Disorder property of the affected region is quantified through calculating the disorder score of each involved amino acid using spine-D (Zhang et al. 2012).

##### Solvent accessible surface areas (ASA)

Solvent accessible surface area has been used as an important feature for variant prioritization (Folkman et al. 2015; Zhao et al. 2013). Based on ASA value, an amino acid can be classified as buried inside or on the surface of a protein. To some degree, ASA can be used to infer the flexibility and predict binding induced structure conformational change of monomeric proteins (Marsh and Teichmann 2011). The ASA value for the affected exon is calculated using Spline-X with default parameters (Faraggi et al. 2009).

##### Protein secondary structure

The most probable secondary structure (alpha-helix, beta sheet, or random coil) on the affected exons are calculated using Spline-X (Faraggi et al. 2009) using default parameters.

##### Overlapping with known or predicted protein family domains (Pfam)

The functional regions of proteins are generally termed as domains. The direct consequence of abnormal splicing is loss or gain of one or more protein domains due to missing or addition of a fragment of protein sequence. The integrity of protein function is determined by the combination of domains and therefore abnormal splicing directly affects protein’s function. We have collected in total 86,748 high quality Pfam-A protein families (49,991 domains, 28,062 families, 703 Motifs, and 7992 repeats) from Pfam database (Finn et al. 2014). As a measurement of the importance of affected exon on protein domains, we calculated a percentage value as the proportion of affected protein region which overlaps with documented Pfam domains.

##### Post-translational modification sites (PTMs)

Post-translational modifications on amino acids play an important role determining the function and activities of a protein. To evaluate the potential PTM status of the exons containing functional sSNVs, We downloaded 372,456 experimentally verified PTM sites from dbPTM 3.0 database (Lu et al. 2013). Among those PTMs, most common modifications are phosphorylation, ubiquitylation, and acetylation. As a comparison on the density of PTM sites, we calculated the normalized PTM site amount per 100 amino acids on the affected protein region.

### Machine learning model

We discovered the excellent classification capability of random forest in our previous study. In addition, in this study, we continued to use random forest as the tool to learn the distinct genomic and protein structural and functional features between disease-causing and neutral variants. Random forest is composed a certain number of decision trees and the final prediction is the polled vote of each tree’s prediction result. For training purpose, random forest algorithm randomly selects (bootstrap) a proportion of training samples for growing each node. The feature for each node is selected from a subset of features bootstrapped from the total set of features, based on a certain split criterion such as information gain or Gini index. In our study, we used a software package called Weka to build our random forest model (Witten et al. 2016). We did not implement feature selection before training our model due to the bootstrap step in both selecting training sample and selecting features when building each node. We tuned the number of trees to grow for random forest as 51 and the number of features subset for building each node as 35. Two different models are trained independently for variants on splicing sites and off-splicing sites, respectively.

## Electronic supplementary material

Below is the link to the electronic supplementary material.

Supplementary material 1 (JPEG 331 kb) Figure S1: The overall distribution of categories of HGMD mutation data set. (A) Out of 697 VSS HGMD variants, 656 (94.1%) are in the DM (disease-causing) category, 30 (4.3%) are in the DM? (likely disease-causing) category, 8 (1.1%) are the FP category (Polymorphism affecting the structure, function or expression of a gene but with no disease association reported yet), and only 3 (0.43%) are from DP or DFP categories (disease-associated). (B) Out of 414 VIE HGMD variants, 344 (83.1%) are from DM (disease-causing) category, 41 (9.9%) are from DM? (likely disease-causing) category, 19 (4.6%) are from FP category, and only 10 (2.42%) are from DP, DFP categories (disease-associated)

Supplementary material 2 (JPEG 329 kb)

Supplementary material 3 (PDF 1985 kb) Figure S2: Heat map of the relative proportion of sSNVs that change RBP binding between disease-causing sSNVs and neutral sSNVs. Each cell, corresponding to one disease–RBP pair, represents the log2-transformed ratio of the proportion of disease-causing sSNVs that change RBP-binding affinity (posterior probability > 0.5), and the proportion of neutral sSNVs. Only significant (P < 0.05) disease–RBP pairs are plotted. Red dots indicate significantly higher proportions of disease-causing sSNVs potentially changing RBP binding than neutral sSNVs, and blue dots indicate lower proportions

Supplementary material 4 (PDF 104 kb) Figure S3: Average binding score changes that are introduced by the disease-causing and neutral variants for each RNA-binding protein. Each dot represents one RNA-binding protein. X- and Y-axes are the average binding score changes induced by the sSNVs in HGMD, and 1000 Genomes databases, respectively. (a) For variants on consensus splice sites, 179 RBPs (red dots) have larger binding score change introduced by disease-causing variants than neutral variants. 22 RBPs (blue dots) have larger binding score change introduced by neutral variants than disease-causing variants. (b) For variants in internal exon, 167 RBPs (red dots) have larger binding score change introduced by disease-causing variants than neutral variants. 34 RBPs (blue dots) have larger binding score change introduced by neutral variants than disease-causing variants

Supplementary material 5 (PDF 335 kb) Figure S4: Cumulative probability density plots for genomic features. Red curves represent HGMD data set and black curves represent 1000 Genomes data set. (a) probability of single strandness for local RNA 2nd structure around variant; (b) RNA 2nd structure change due to variant; (c) matching score of acceptor site; (d) matching score of donor site; (e) difference of matching score due to mutation, either on acceptor site or donor site; (f) change of exon splicing motif density defined as cluster score; (g) cluster score of original exon sequence;(h) proximity to donor site or acceptor site; (i) upstream intron length; (j) exon length; (k) downstream intron length; (l) average PhyloP score of ± 7 bp around SNP locus; (m) max matching score of SFRS1 on wild exon sequence; (n) max matching score of SFRS1 on mutated exon sequence; (o) max matching score of SFRS2 on wild sequence; (p) max matching score of SFRS2 on mutated sequence; (q) max matching score of SFRS5 on wild sequence; (r) max matching score of SFRS5 on mutated sequence; (s) max matching score of SFRS6 on wild sequence; (t) max matching score of SFRS6 on mutated sequence

Supplementary material 6 (PDF 86 kb) Figure S5: Cumulative probability density plots for accessible surface area features (ASA). Red curves represent HGMD data set and black curves represent 1000 Genomes data set. (a) average ASA of all amino acids; (b) min ASA of all amino acids (c) max ASA of all amino acids

Supplementary material 7 (PDF 258 kb) Figure S6: Cumulative probability density plots for disorder scores. Red curves represent HGMD data set and black curves represent 1000 Genomes data set. (a) min disorder score of all amino acids; (b) max disorder score of all amino acids; (c) average disorder score of amino acids in disordered region; (d) average disorder score of amino acids in structured region; (e) number of switchings between disorder region and structured region; (f) average disorder region length; (g) average structure region length; (h) max disorder region length; (i) min disorder region length; (j) max structured region length; (k) min structured region length; (l) average disorder score of all amino acids

Supplementary material 8 (PDF 95 kb) Figure S7: Cumulative probability density plots for Pfam and post-translational modification (PTM) features. Red curves represent HGMD data set and black curves represent 1000 Genomes data set. (a) Percentage of exon length overlapped with Pfam domains (b) normalized PTM sites count per 100 amino acids

Supplementary material 9 (PDF 255 kb) Figure S8: Cumulative probability density plots for secondary structure (SS) score features. Red curves represent HGMD data set and black curves represent 1000 Genomes data set. (a) max probability of predicted structure of all amino acids; (b) min probability of predicted structure of all amino acids; (c) average probability of predicted structure of all amino acids; (d) average probability of amino acids in beta sheet; (e) min probability of amino acids in beta sheet; (f) max probability of amino acids in beta sheet; (g) average probability of amino acids in random coil; (h) min probability of amino acids in random coil; (i) max probability of amino acids in random coil (j) average probability of amino acids in alpha-helix; (k) min probability of amino acids in alpha-helix; (l) max probability of amino acids in alpha-helix

Supplementary material 10 (PDF 133 kb) Figure S9: Evaluation of classification power of each type of feature categories. (a) ROC curves for VSS models built using all features based on tenfold cross validation (red, AUC = 0.83), DNA-nucleotide conservation phylop score (black, AUC = 0.56), splicing regulation features (blue, AUC = 0.91) and protein features (magenta, AUC = 0.67). (b) ROC curves for VIE models built using all features based on tenfold cross validation (red, AUC = 0.86), DNA-nucleotide conservation phylop score (black, AUC = 0.59), splicing regulation features (blue, AUC = 0.81), and protein features (magenta, AUC = 0.71)

Supplementary material 11 (PDF 120 kb)

Supplementary material 12 (PDF 64 kb) Figure S10: Performance of models built using data after removing homologous genes. (A) VSS models built using variants from the gene selected out of one gene family. The area under curve is 0.93. (B) VIE models built using variants from the gene selected out of one gene family. The area under curve is 0.86

Supplementary material 13 (PDF 76 kb)

Supplementary material 14 (DOCX 65 kb)
