# Supplementary figures and images for: regSNPs-splicing: a tool for prioritizing synonymous single-nucleotide substitution
Source: Hum Genet. 2017 Apr 8;136(9):1279–89. doi: 10.1007/s00439-017-1783-x (PMC5602096; doi:10.1007/s00439-017-1783-x)

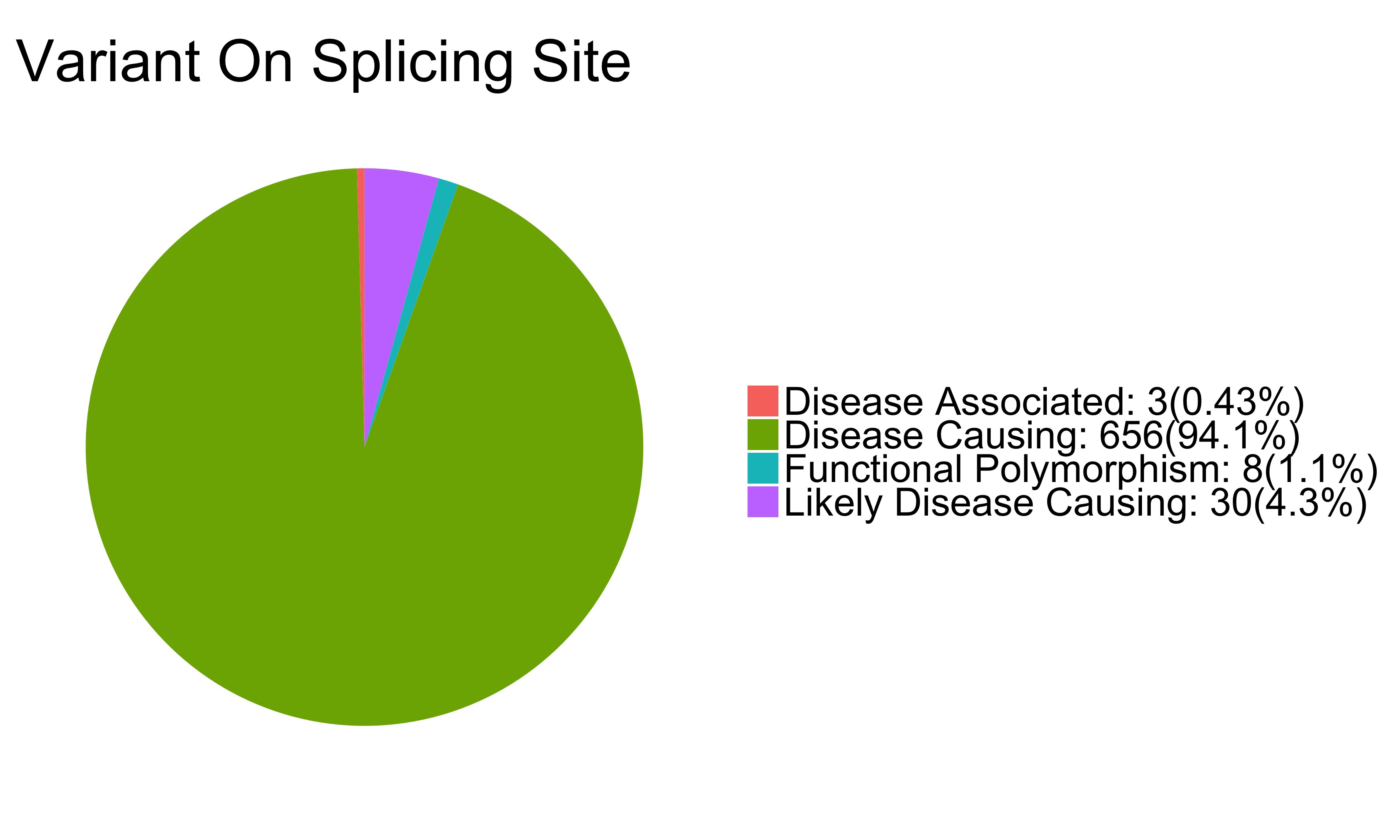

Supplement: Supplementary file 1 — Supplementary material 1 (JPEG 331 kb) Figure S1: The overall distribution of categories of HGMD mutation data set. (A) Out of 697 VSS HGMD variants, 656 (94.1%) are in the DM (disease-causing) category, 30 (4.3%) are in the DM? (likely disease-causing) category, 8 (1.1%) are the FP category (Polymorphism affecting the structure, function or expression of a gene but with no disease association reported yet), and only 3 (0.43%) are from DP or DFP categories (disease-associated). (B) Out of 414 VIE HGMD variants, 344 (83.1%) are from DM (disease-causing) category, 41 (9.9%) are from DM? (likely disease-causing) category, 19 (4.6%) are from FP category, and only 10 (2.42%) are from DP, DFP categories (disease-associated) [file 439_2017_1783_MOESM1_ESM.jpeg]

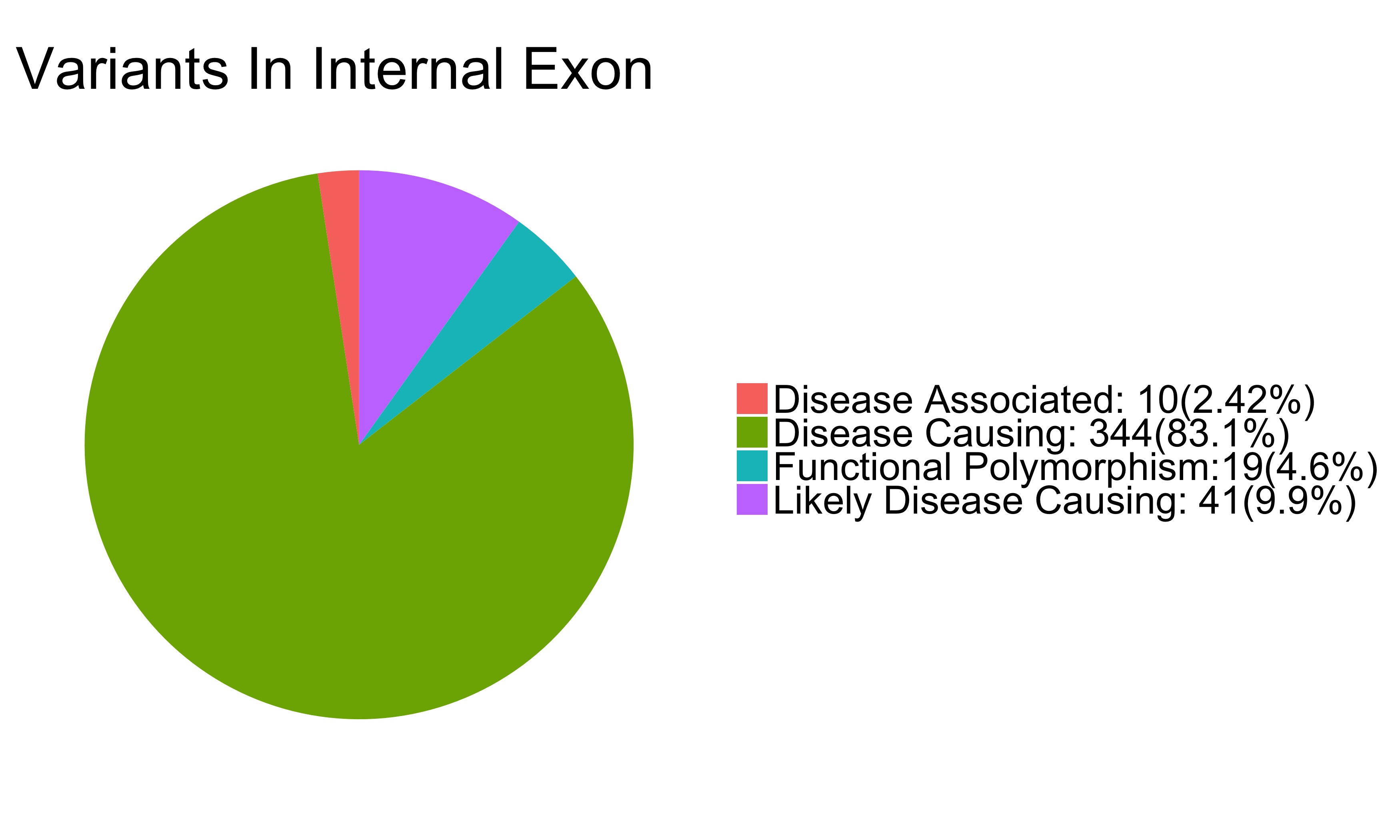

Supplement: Supplementary file 2 — Supplementary material 2 (JPEG 329 kb) [file 439_2017_1783_MOESM2_ESM.jpeg]

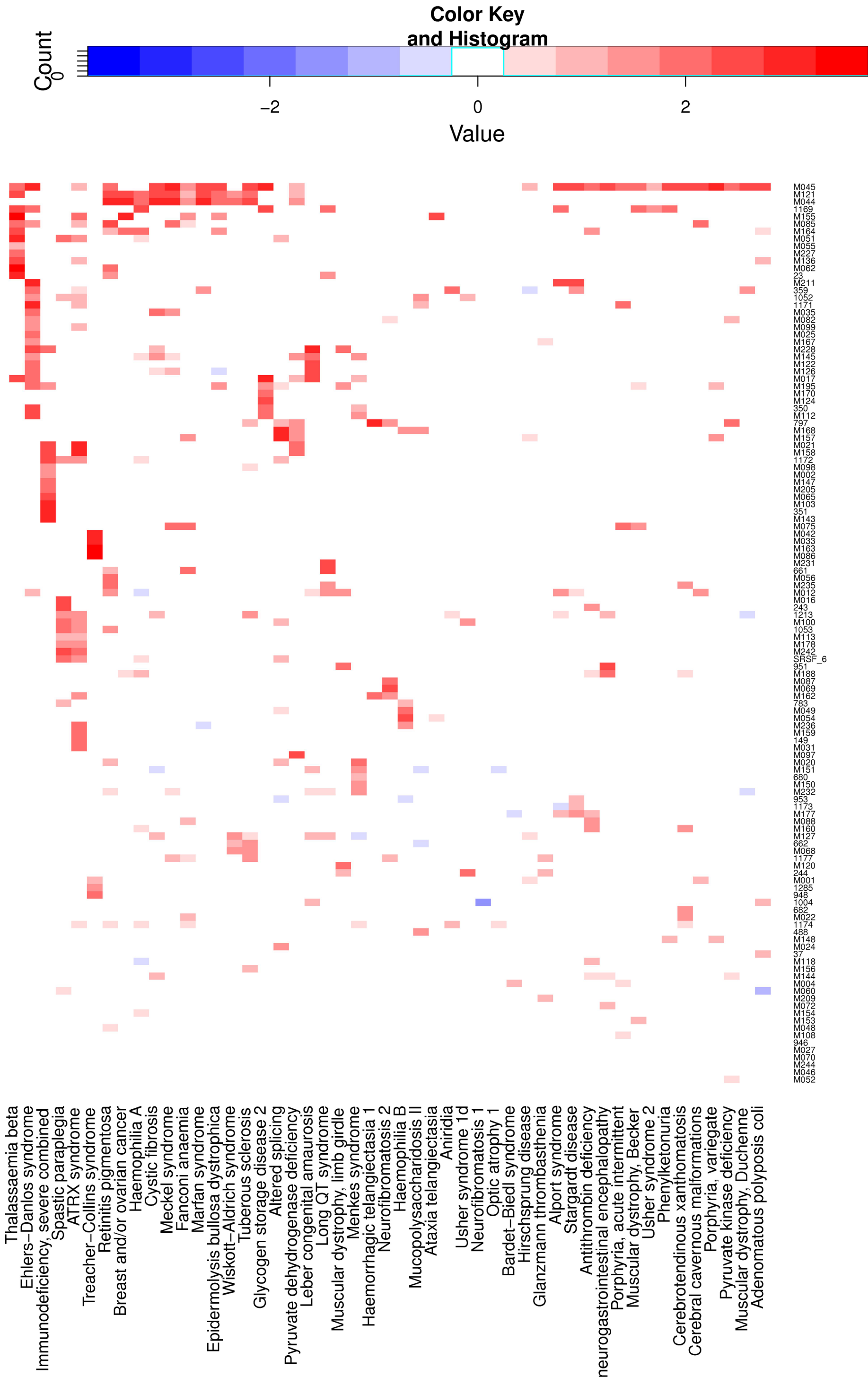

Supplement: Supplementary file 3 — Supplementary material 3 (PDF 1985 kb) Figure S2: Heat map of the relative proportion of sSNVs that change RBP binding between disease-causing sSNVs and neutral sSNVs. Each cell, corresponding to one disease–RBP pair, represents the log2-transformed ratio of the proportion of disease-causing sSNVs that change RBP-binding affinity (posterior probability > 0.5), and the proportion of neutral sSNVs. Only significant (P < 0.05) disease–RBP pairs are plotted. Red dots indicate significantly higher proportions of disease-causing sSNVs potentially changing RBP binding than neutral sSNVs, and blue dots indicate lower proportions [file 439_2017_1783_MOESM3_ESM.pdf]

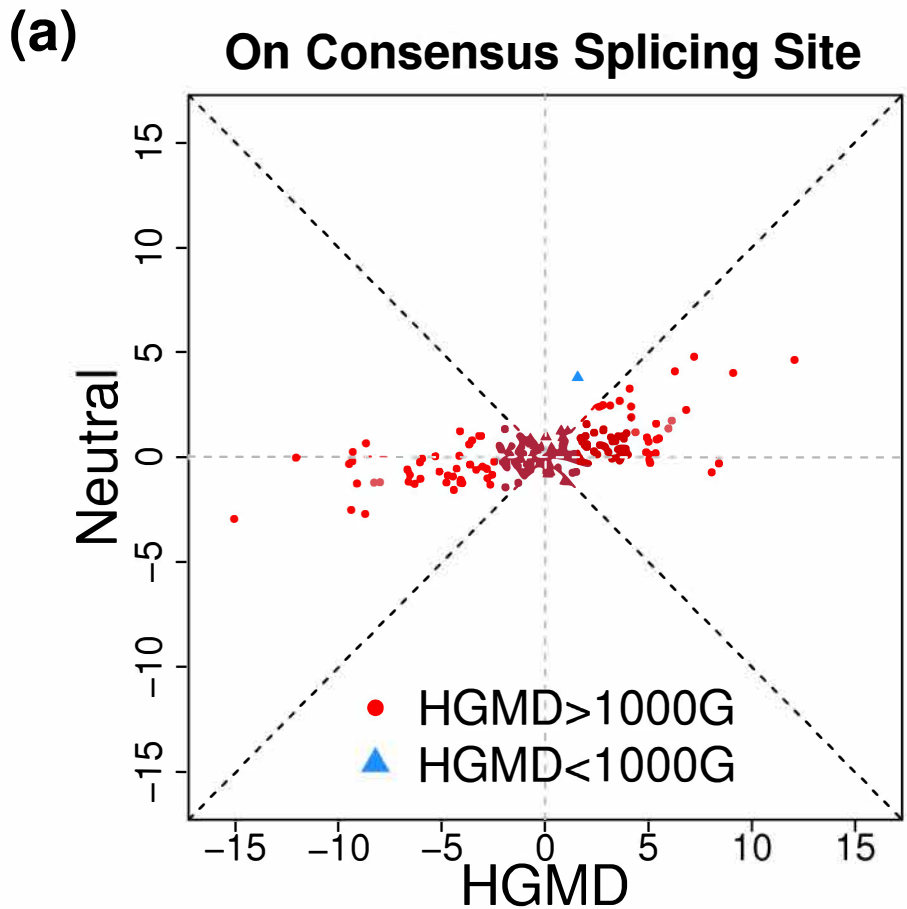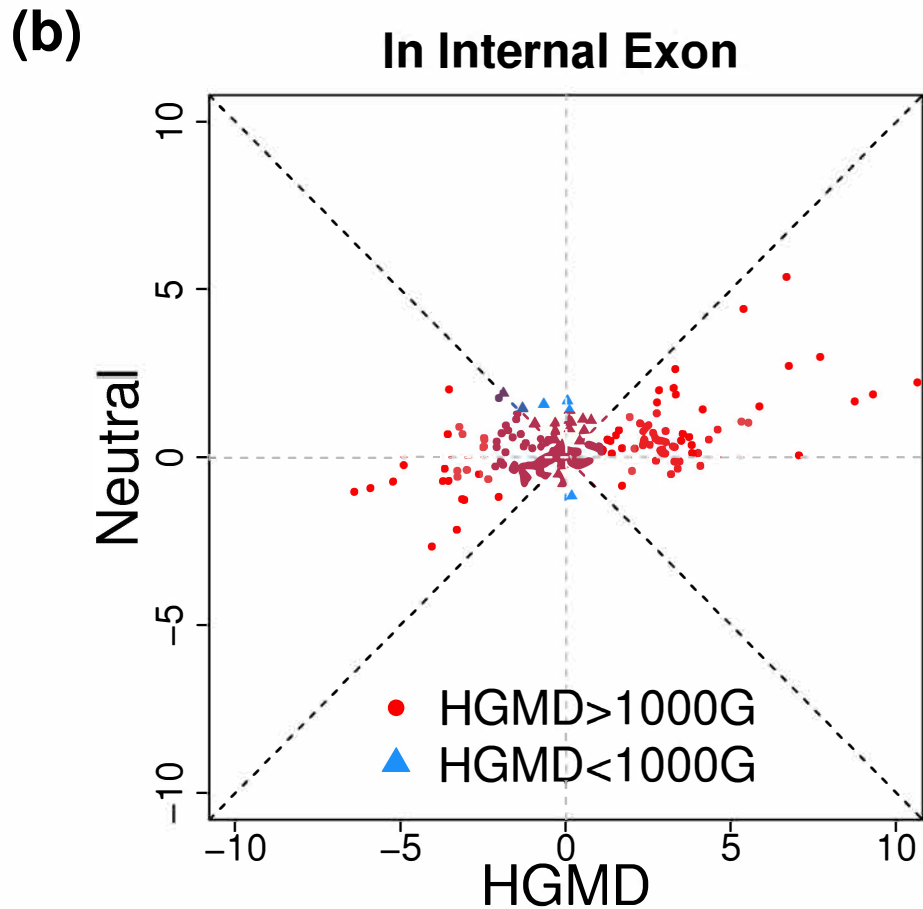

Supplement: Supplementary file 4 — Supplementary material 4 (PDF 104 kb) Figure S3: Average binding score changes that are introduced by the disease-causing and neutral variants for each RNA-binding protein. Each dot represents one RNA-binding protein. X- and Y-axes are the average binding score changes induced by the sSNVs in HGMD, and 1000 Genomes databases, respectively. (a) For variants on consensus splice sites, 179 RBPs (red dots) have larger binding score change introduced by disease-causing variants than neutral variants. 22 RBPs (blue dots) have larger binding score change introduced by neutral variants than disease-causing variants. (b) For variants in internal exon, 167 RBPs (red dots) have larger binding score change introduced by disease-causing variants than neutral variants. 34 RBPs (blue dots) have larger binding score change introduced by neutral variants than disease-causing variants [file 439_2017_1783_MOESM4_ESM.pdf]

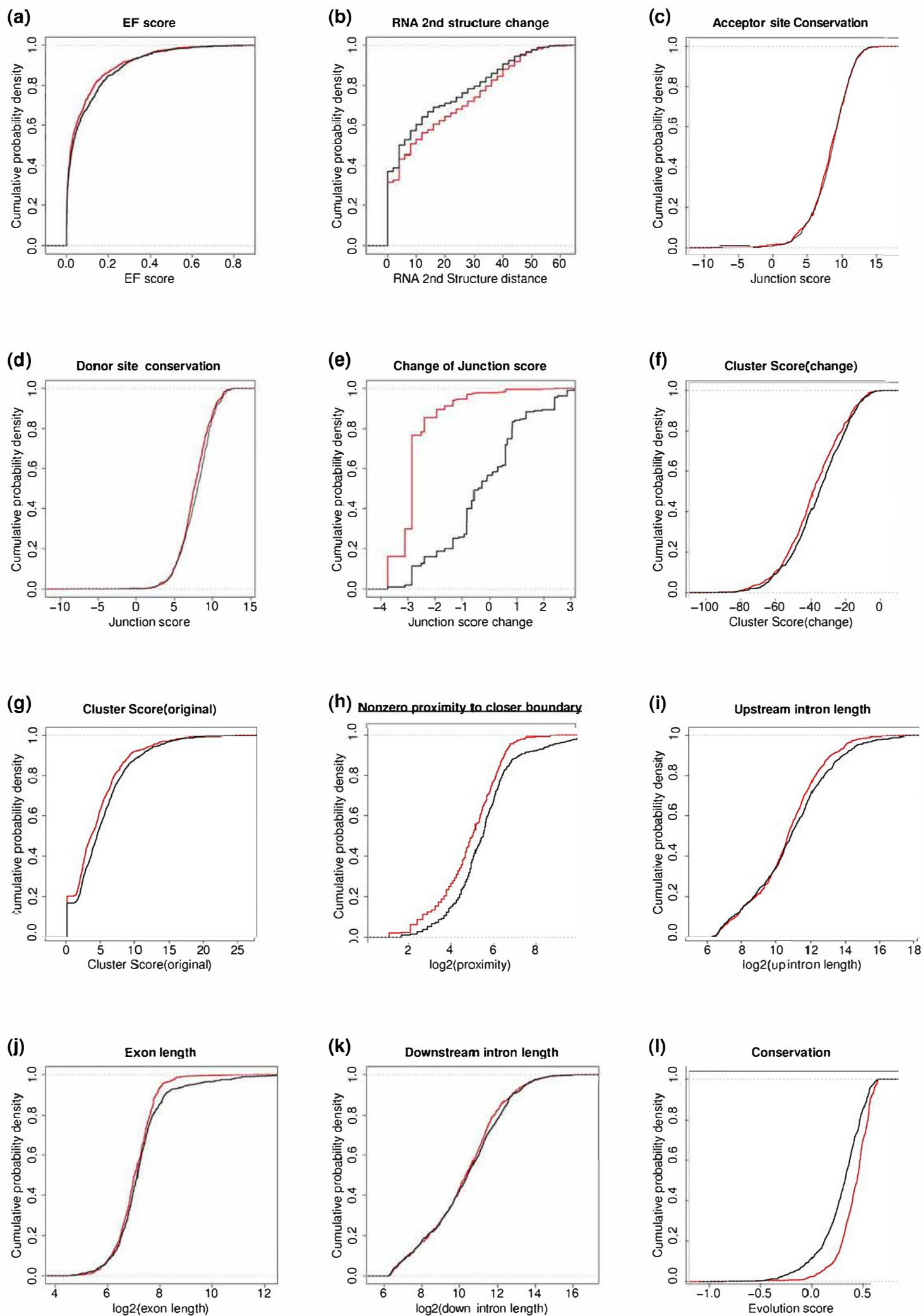

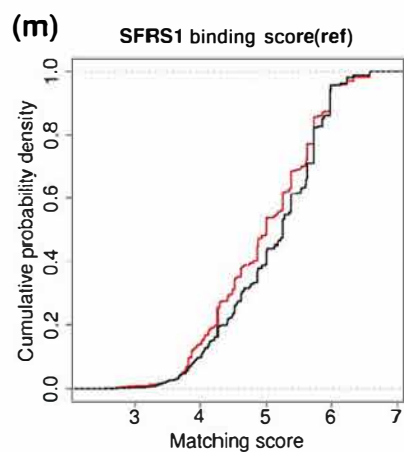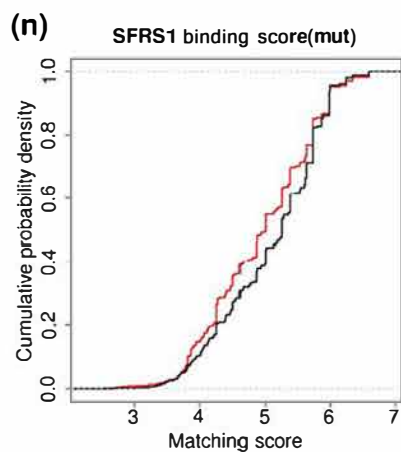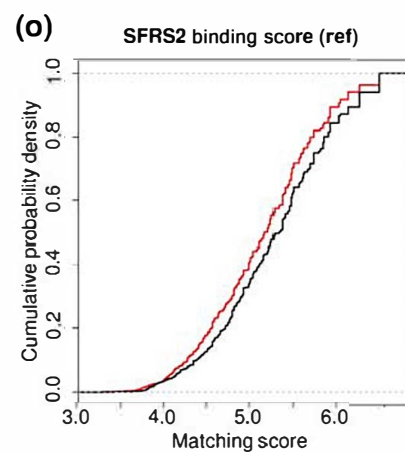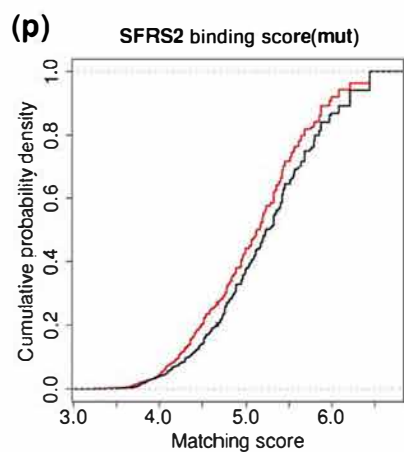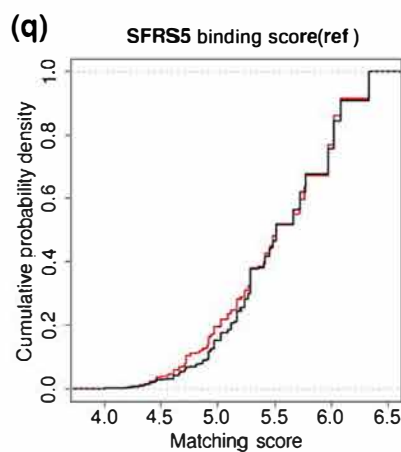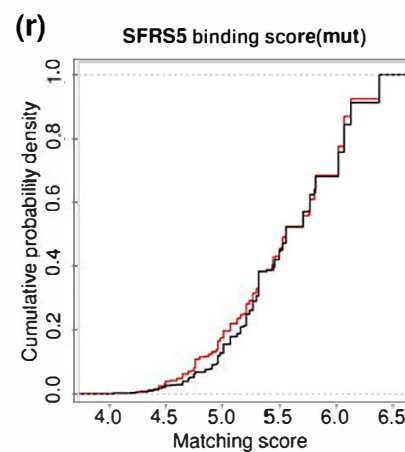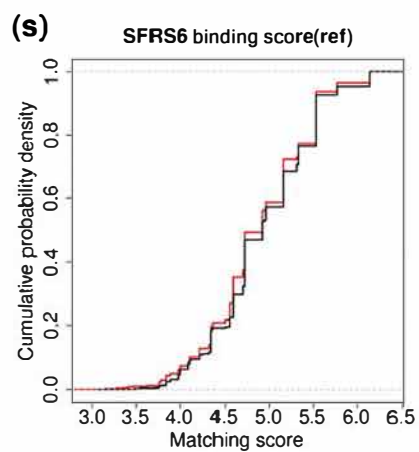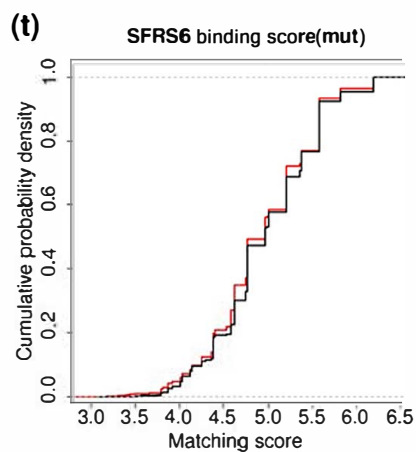

Supplement: Supplementary file 5 — Supplementary material 5 (PDF 335 kb) Figure S4: Cumulative probability density plots for genomic features. Red curves represent HGMD data set and black curves represent 1000 Genomes data set. (a) probability of single strandness for local RNA 2nd structure around variant; (b) RNA 2nd structure change due to variant; (c) matching score of acceptor site; (d) matching score of donor site; (e) difference of matching score due to mutation, either on acceptor site or donor site; (f) change of exon splicing motif density defined as cluster score; (g) cluster score of original exon sequence;(h) proximity to donor site or acceptor site; (i) upstream intron length; (j) exon length; (k) downstream intron length; (l) average PhyloP score of ± 7 bp around SNP locus; (m) max matching score of SFRS1 on wild exon sequence; (n) max matching score of SFRS1 on mutated exon sequence; (o) max matching score of SFRS2 on wild sequence; (p) max matching score of SFRS2 on mutated sequence; (q) max matching score of SFRS5 on wild sequence; (r) max matching score of SFRS5 on mutated sequence; (s) max matching score of SFRS6 on wild sequence; (t) max matching score of SFRS6 on mutated sequence [file 439_2017_1783_MOESM5_ESM.pdf]

**(a)**

Average ASA Score

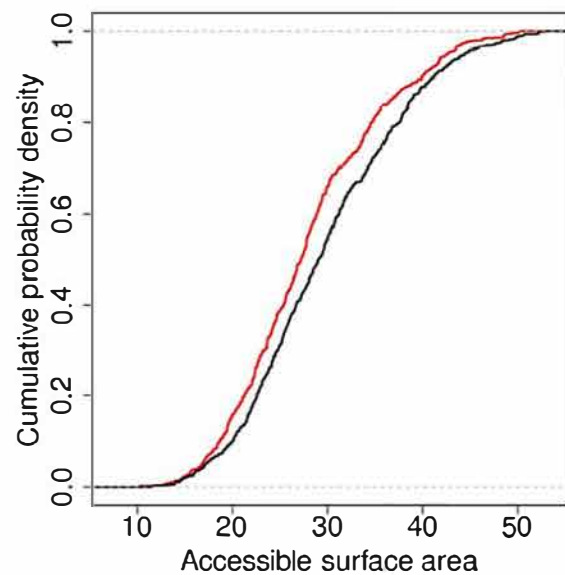

**(b)**

Min ASA Score

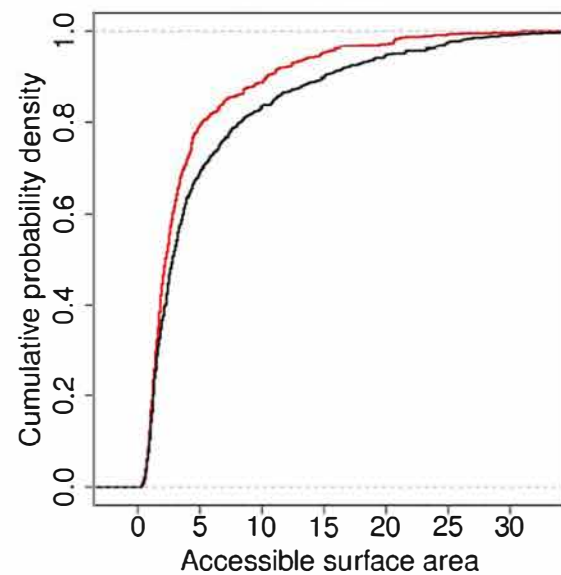

**(c)**

Max ASA Score

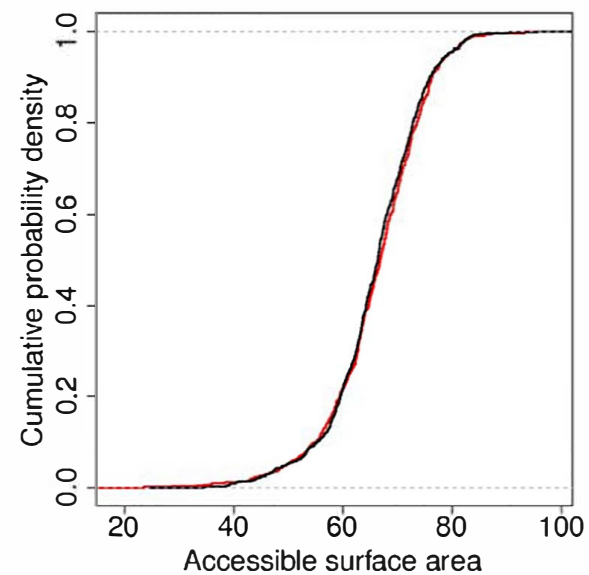

Supplement: Supplementary file 6 — Supplementary material 6 (PDF 86 kb) Figure S5: Cumulative probability density plots for accessible surface area features (ASA). Red curves represent HGMD data set and black curves represent 1000 Genomes data set. (a) average ASA of all amino acids; (b) min ASA of all amino acids (c) max ASA of all amino acids [file 439_2017_1783_MOESM6_ESM.pdf]

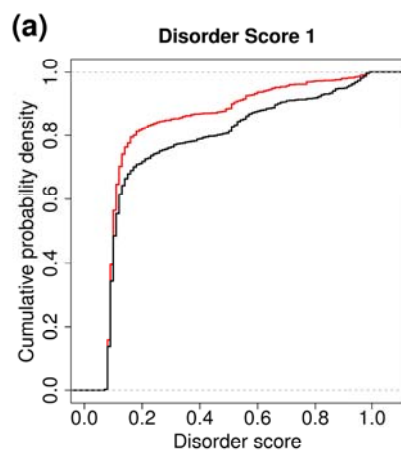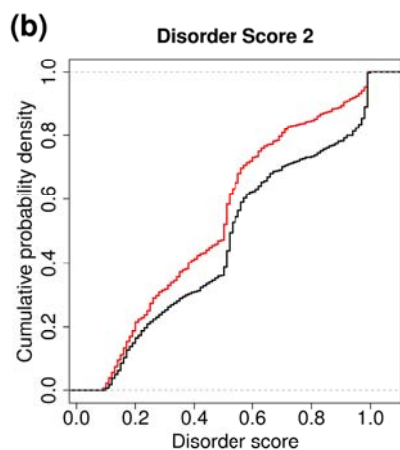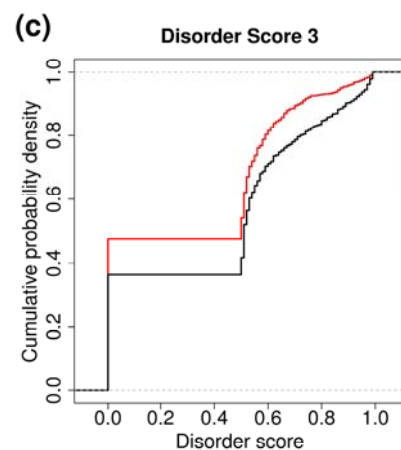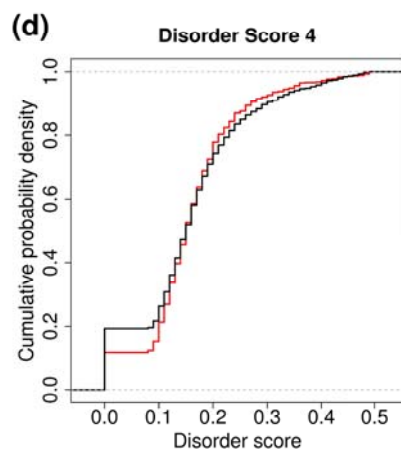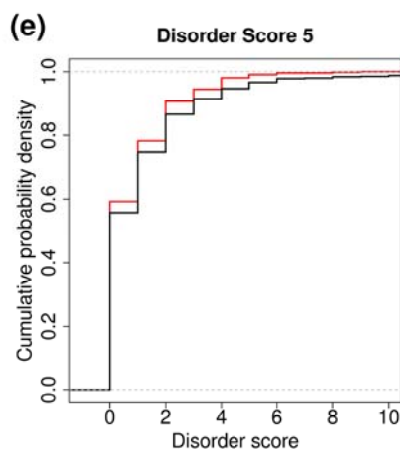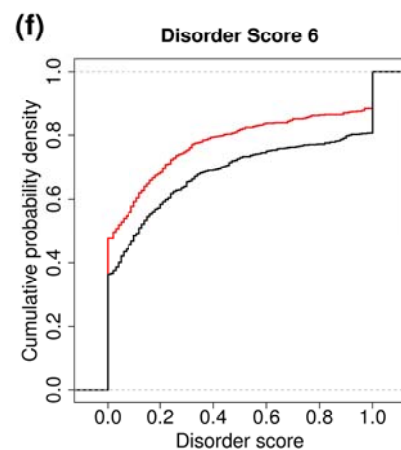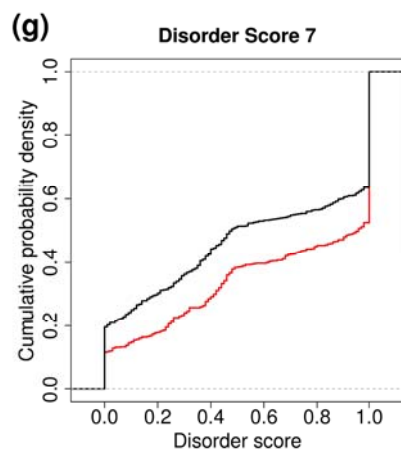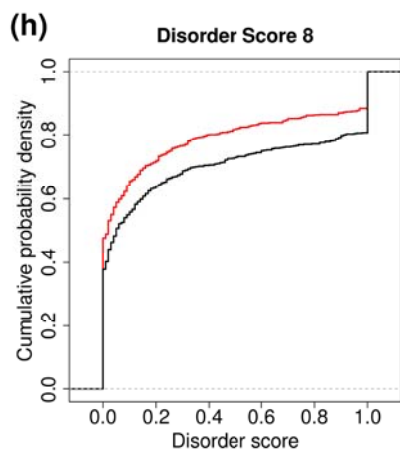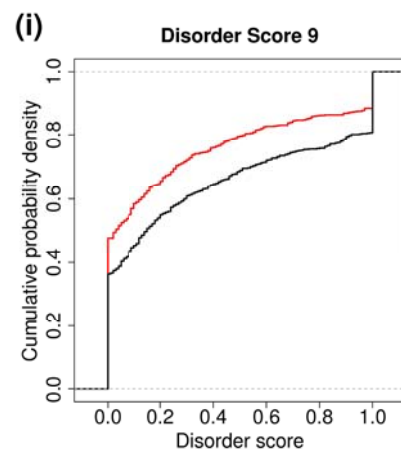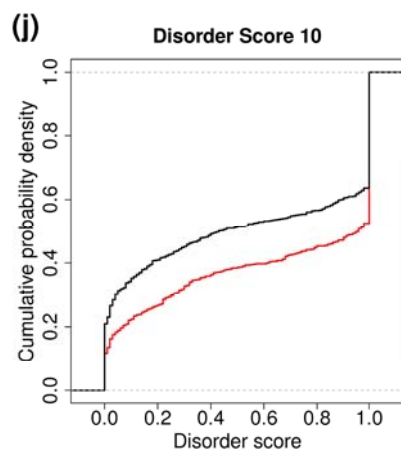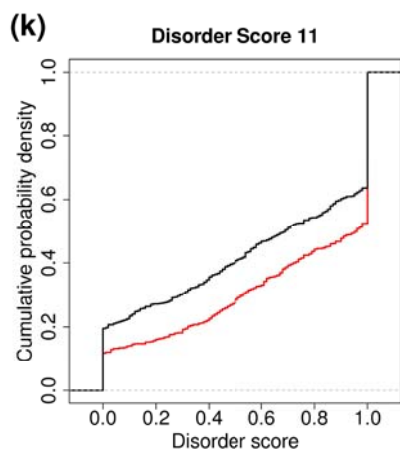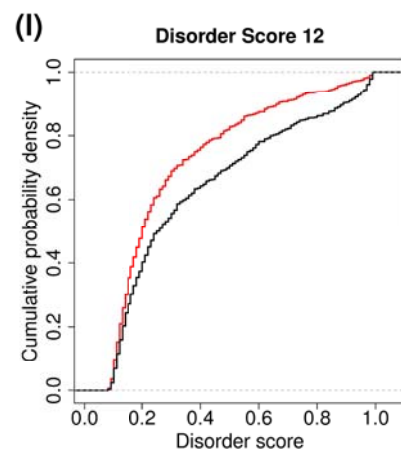

Supplement: Supplementary file 7 — Supplementary material 7 (PDF 258 kb) Figure S6: Cumulative probability density plots for disorder scores. Red curves represent HGMD data set and black curves represent 1000 Genomes data set. (a) min disorder score of all amino acids; (b) max disorder score of all amino acids; (c) average disorder score of amino acids in disordered region; (d) average disorder score of amino acids in structured region; (e) number of switchings between disorder region and structured region; (f) average disorder region length; (g) average structure region length; (h) max disorder region length; (i) min disorder region length; (j) max structured region length; (k) min structured region length; (l) average disorder score of all amino acids [file 439_2017_1783_MOESM7_ESM.pdf]

**(a)** Overlap with Pfam domains

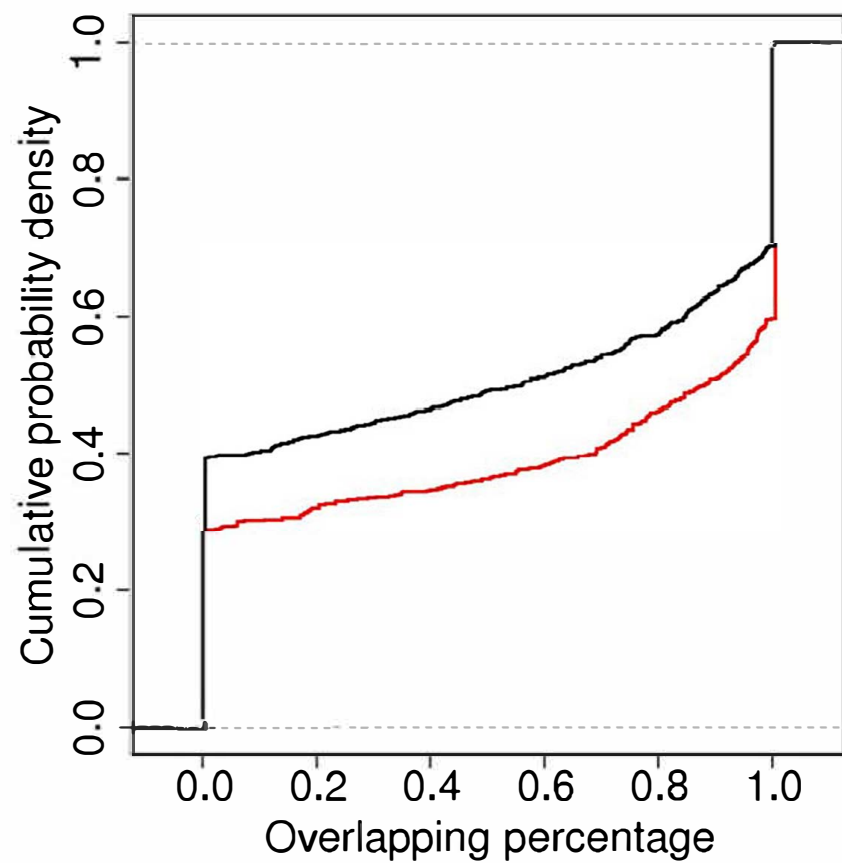

**(b)** PTM density

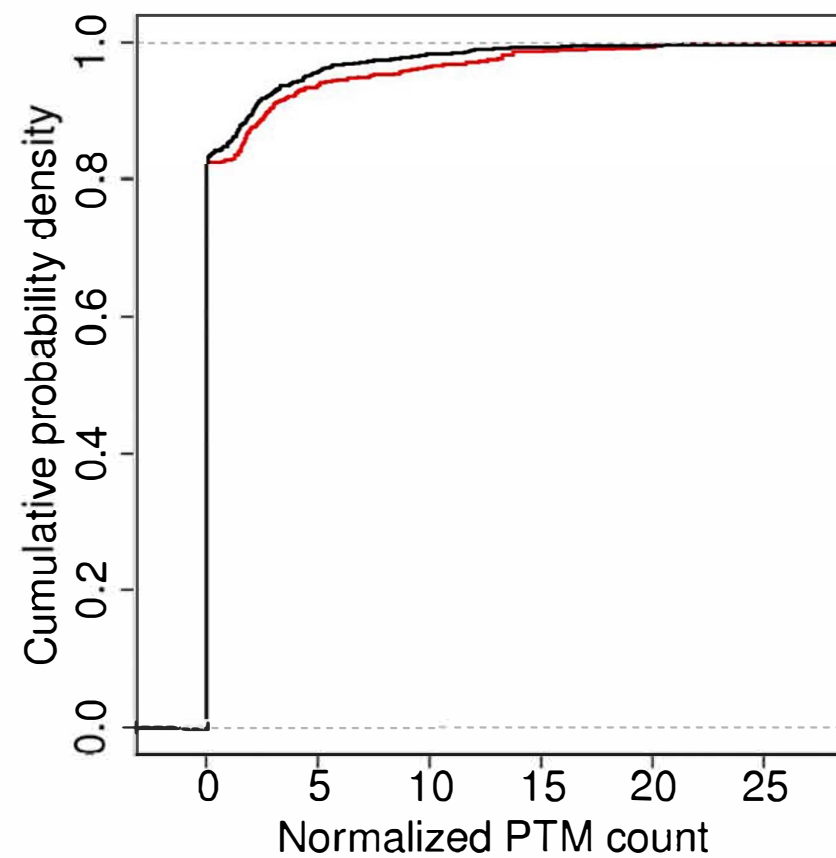

Supplement: Supplementary file 8 — Supplementary material 8 (PDF 95 kb) Figure S7: Cumulative probability density plots for Pfam and post-translational modification (PTM) features. Red curves represent HGMD data set and black curves represent 1000 Genomes data set. (a) Percentage of exon length overlapped with Pfam domains (b) normalized PTM sites count per 100 amino acids [file 439_2017_1783_MOESM8_ESM.pdf]

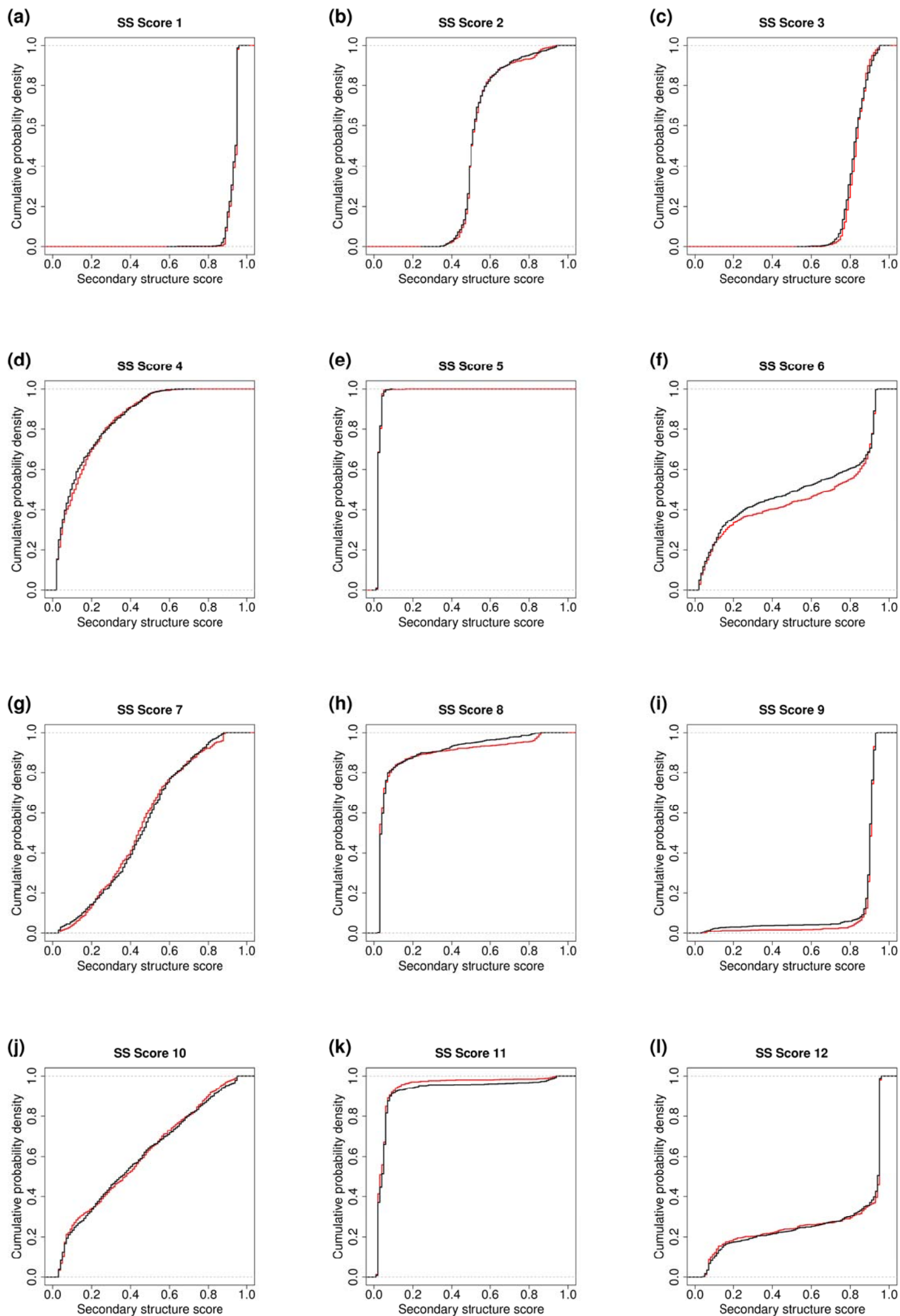

Supplement: Supplementary file 9 — Supplementary material 9 (PDF 255 kb) Figure S8: Cumulative probability density plots for secondary structure (SS) score features. Red curves represent HGMD data set and black curves represent 1000 Genomes data set. (a) max probability of predicted structure of all amino acids; (b) min probability of predicted structure of all amino acids; (c) average probability of predicted structure of all amino acids; (d) average probability of amino acids in beta sheet; (e) min probability of amino acids in beta sheet; (f) max probability of amino acids in beta sheet; (g) average probability of amino acids in random coil; (h) min probability of amino acids in random coil; (i) max probability of amino acids in random coil (j) average probability of amino acids in alpha-helix; (k) min probability of amino acids in alpha-helix; (l) max probability of amino acids in alpha-helix [file 439_2017_1783_MOESM9_ESM.pdf]

## On Consensus Splicing Site

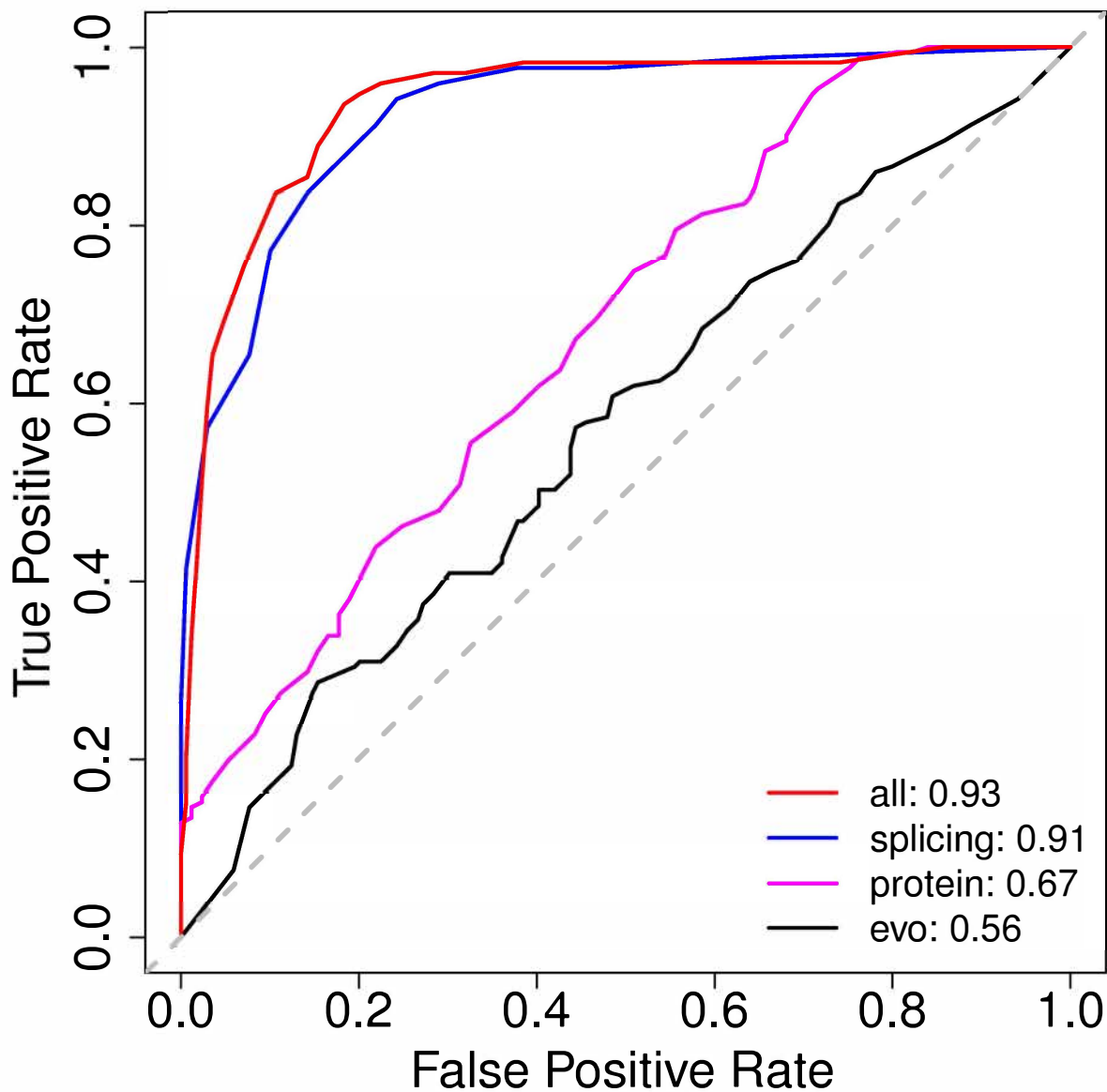

Supplement: Supplementary file 10 — Supplementary material 10 (PDF 133 kb) Figure S9: Evaluation of classification power of each type of feature categories. (a) ROC curves for VSS models built using all features based on tenfold cross validation (red, AUC = 0.83), DNA-nucleotide conservation phylop score (black, AUC = 0.56), splicing regulation features (blue, AUC = 0.91) and protein features (magenta, AUC = 0.67). (b) ROC curves for VIE models built using all features based on tenfold cross validation (red, AUC = 0.86), DNA-nucleotide conservation phylop score (black, AUC = 0.59), splicing regulation features (blue, AUC = 0.81), and protein features (magenta, AUC = 0.71) [file 439_2017_1783_MOESM10_ESM.pdf]

## In Internal Exon

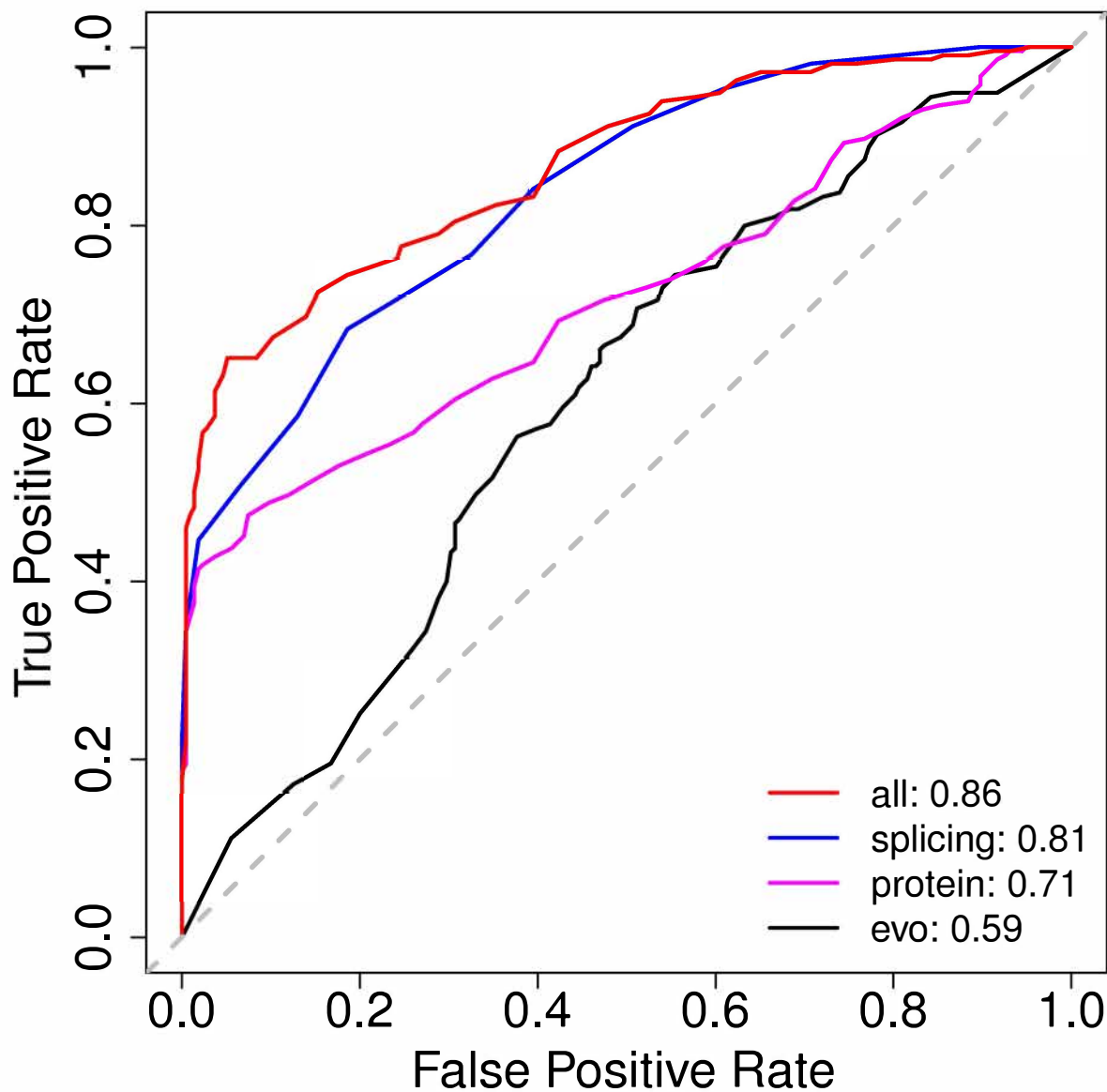

Supplement: Supplementary file 11 — Supplementary material 11 (PDF 120 kb) [file 439_2017_1783_MOESM11_ESM.pdf]

## On ConSensus Splicing Site

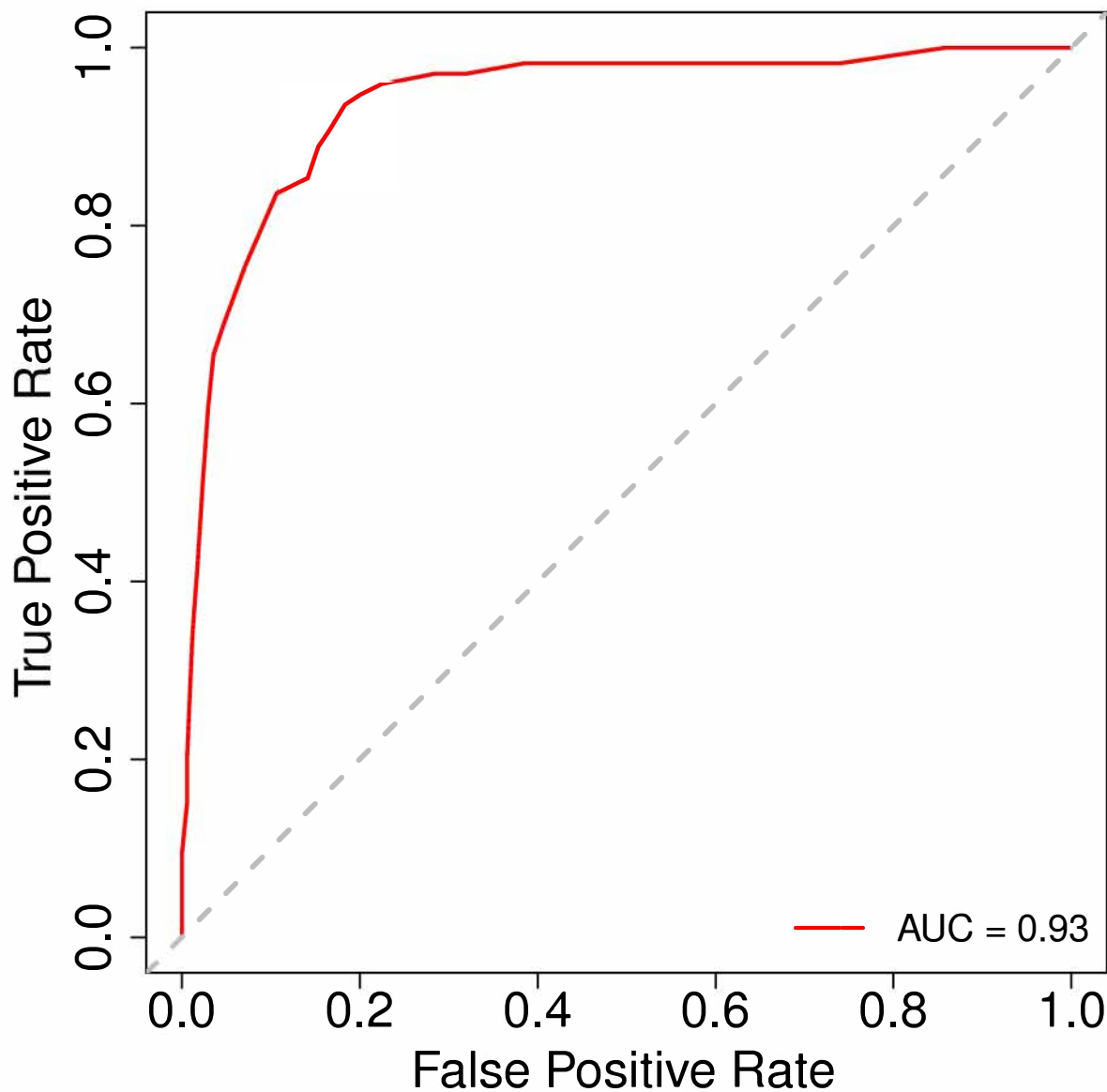

Supplement: Supplementary file 12 — Supplementary material 12 (PDF 64 kb) Figure S10: Performance of models built using data after removing homologous genes. (A) VSS models built using variants from the gene selected out of one gene family. The area under curve is 0.93. (B) VIE models built using variants from the gene selected out of one gene family. The area under curve is 0.86 [file 439_2017_1783_MOESM12_ESM.pdf]

## In Internal Exon

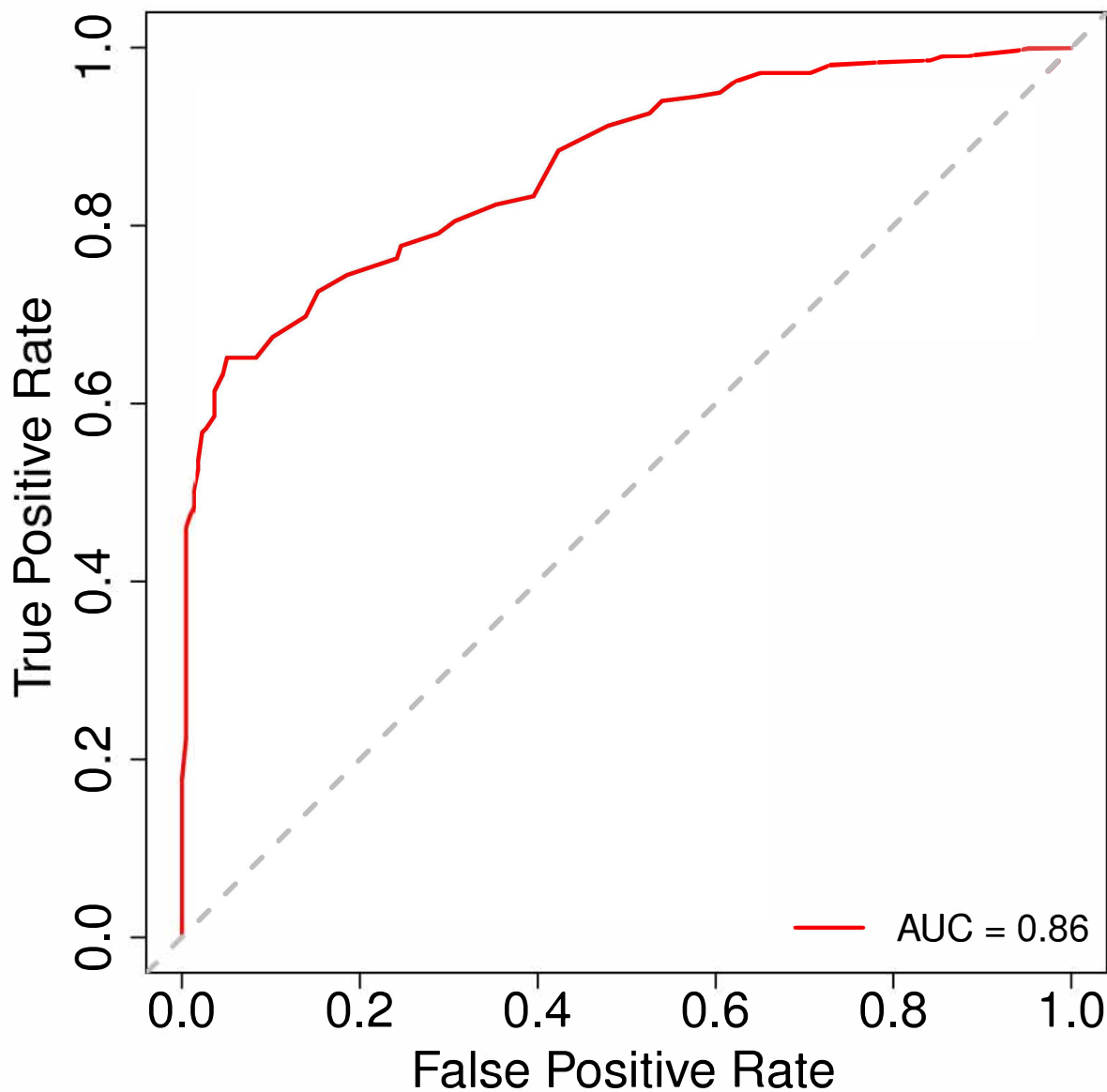

Supplement: Supplementary file 13 — Supplementary material 13 (PDF 76 kb) [file 439_2017_1783_MOESM13_ESM.pdf]
