# Supplementary material for: regSNPs-splicing: a tool for prioritizing synonymous single-nucleotide substitution
Source: Hum Genet. 2017 Apr 8;136(9):1279–89. doi: 10.1007/s00439-017-1783-x (PMC5602096; doi:10.1007/s00439-017-1783-x)
Supplement: Supplementary file 14 — Supplementary material 14 (DOCX 65 kb) [file 439_2017_1783_MOESM14_ESM.docx]

**Supplement Table 1: List of all of the features utilized in this study.**

| **Feature** | **D value (K-S test)** | **P-value (K-S test)** | **MCC (on_ss)** | **MCC (off_ss)** | **average  MCC** | **Feature description** |
| --- | --- | --- | --- | --- | --- | --- |
| exon_intron_len_1 | 0.082 | 0.012 | 0.003 | 0.117 | 0.060 | upstream intron length |
| exon_intron_len_2 | 0.082 | 0.012 | 0.105 | 0.233 | 0.169 | exon length |
| exon_intron_len_3 | 0.056 | 0.183 | 0.078 | 0.140 | 0.109 | downstream intron length |
| junction_score_1 | 0.041 | 0.548 | 0.053 | 0.147 | 0.100 | matching score of acceptor site |
| junction_score_2 | 0.088 | 0.005 | 0.046 | 0.160 | 0.103 | matching score of donor site |
| junction_score_ change | 0.712 | 0.000 | 0.701 | NA | 0.701 | change of either matching score |
| sfrs1_3 | 0.101 | 0.001 | 0.073 | 0.146 | 0.110 | max matching score of SFRS1 on ref sequence |
| sfrs1_5 | 0.112 | 0.000 | 0.092 | 0.143 | 0.117 | max matching score of SFRS1 on mut sequence |
| sfrs2_3 | 0.087 | 0.006 | 0.123 | 0.165 | 0.144 | max matching score of SFRS2 on ref sequence |
| sfrs2_5 | 0.084 | 0.009 | 0.123 | 0.165 | 0.144 | max matching score of SFRS2 on mut sequence |
| sfrs5_3 | 0.051 | 0.270 | 0.036 | 0.090 | 0.063 | max matching score of SFRS5 on ref sequence |
| sfrs5_5 | 0.049 | 0.330 | 0.000 | 0.078 | 0.039 | max matching score of SFRS5 on mut sequence |
| sfrs6_3 | 0.055 | 0.201 | 0.106 | 0.148 | 0.127 | max matching score of SFRS6 on ref sequence |
| sfrs6_5 | 0.048 | 0.334 | 0.098 | 0.148 | 0.123 | max matching score of SFRS6 on mut sequence |
| proximity_acceptor | 0.105 | 0.052 | NA | 0.174 | 0.174 | proximity to acceptor site |
| proximity_donor | 0.123 | 0.014 | NA | 0.232 | 0.232 | proximity to donor site |
| evolution | 0.270 | 0.000 | 0.306 | 0.243 | 0.274 | average PhyloP score of +/- 7bp of SNP locus |
| cluster_score_ change | 0.093 | 0.003 | 0.028 | 0.174 | 0.101 | change of cluster score |
| cluster_score_ref | 0.106 | 0.000 | 0.150 | 0.209 | 0.180 | cluster score of ref sequence |
| pfam_coverage | 0.139 | 0.000 | 0.125 | 0.109 | 0.117 | %exon overlapped with Pfams |
| ptm_1 | 0.038 | 0.635 | 0.079 | 0.039 | 0.059 | normalized PTM sites per 100 amino acids |
| disorder_score_1 | 0.106 | 0.000 | 0.189 | 0.122 | 0.155 | min disorder score of all amino acids |
| disorder_score_2 | 0.145 | 0.000 | 0.168 | 0.251 | 0.209 | max disorder score of all amino acids |
| disorder_score_3 | 0.130 | 0.000 | 0.164 | 0.212 | 0.188 | average disorder score of amino acids in disordered region |
| disorder_score_4 | 0.076 | 0.023 | 0.121 | 0.112 | 0.117 | average disorder score of amino acids in structured region |
| disorder_score_5 | 0.041 | 0.547 | 0.067 | 0.099 | 0.083 | number of switchings between disorder region and structured region |
| disorder_score_6 | 0.124 | 0.000 | 0.185 | 0.150 | 0.167 | average disorder region length |
| disorder_score_7 | 0.149 | 0.000 | 0.191 | 0.166 | 0.178 | average structure region length |
| disorder_score_8 | 0.110 | 0.000 | 0.185 | 0.155 | 0.170 | max disorder region length |
| disorder_score_9 | 0.135 | 0.000 | 0.182 | 0.140 | 0.161 | min disorder region length |
| disorder_score_10 | 0.146 | 0.000 | 0.174 | 0.156 | 0.165 | max structured region length |
| disorder_score_11 | 0.139 | 0.000 | 0.191 | 0.166 | 0.178 | min structured region length |
| disorder_score_12 | 0.140 | 0.000 | 0.178 | 0.177 | 0.178 | average disorder score of all amino acids |
| ss_1 | 0.053 | 0.237 | 0.102 | 0.093 | 0.097 | max probability of predicted structure of all amino acids |
| ss_2 | 0.032 | 0.839 | 0.040 | 0.114 | 0.077 | min probability of predicted structure of all amino acids |
| ss_3 | 0.082 | 0.011 | 0.080 | 0.136 | 0.108 | average probability of predicted structure of all amino acids |
| ss_4 | 0.064 | 0.091 | 0.072 | 0.029 | 0.050 | average probability of amino acids in beta sheet |
| ss_5 | 0.026 | 0.960 | 0.077 | 0.029 | 0.053 | min probability of amino acids in beta sheet |
| ss_6 | 0.068 | 0.061 | 0.082 | 0.047 | 0.065 | max probability of amino acids in beta sheet |
| ss_7 | 0.049 | 0.316 | 0.021 | 0.112 | 0.066 | average probability of amino acids in random coil |
| ss_8 | 0.058 | 0.152 | 0.072 | 0.093 | 0.082 | min probability of amino acids in random coil |
| ss_9 | 0.073 | 0.034 | 0.125 | 0.030 | 0.078 | max probability of amino acids in random coil |
| ss_10 | 0.031 | 0.843 | 0.036 | 0.093 | 0.065 | average probability of amino acids in alpha-helix |
| ss_11 | 0.067 | 0.063 | 0.109 | 0.046 | 0.077 | min probability of amino acids in alpha-helix |
| ss_12 | 0.053 | 0.237 | 0.070 | 0.029 | 0.050 | max probability of amino acids in alpha-helix |
| asa_1 | 0.128 | 0.000 | 0.145 | 0.151 | 0.148 | average ASA of all amino acids |
| asa_2 | 0.107 | 0.000 | 0.170 | 0.146 | 0.158 | min ASA of all amino acids |
| asa_3 | 0.045 | 0.412 | 0.130 | 0.152 | 0.141 | max ASA of all amino acids |
| EF score | 0.058 | 0.151 | 0.000 | 0.030 | 0.015 | probability of single strandness around variant |
| RNA 2nd structure  change | 0.092 | 0.003 | 0.029 | 0.005 | 0.017 | RNA 2nd structure change due to variant |
| magnitude_1 | 0.066 | 0.074 | 0.306 | 0.243 | 0.274 | 1004_8676391 |
| magnitude_2 | 0.219 | 0.000 | 0.120 | 0.046 | 0.083 | 1052_17318228 |
| magnitude_3 | 0.075 | 0.028 | 0.298 | 0.105 | 0.201 | 1053_17318228 |
| magnitude_4 | 0.219 | 0.000 | 0.088 | 0.160 | 0.124 | 1169_19561594 |
| magnitude_5 | 0.252 | 0.000 | 0.370 | 0.092 | 0.231 | 1170_19561594 |
| magnitude_6 | 0.153 | 0.000 | 0.395 | 0.175 | 0.285 | 1171_19561594 |
| magnitude_7 | 0.116 | 0.000 | 0.335 | 0.233 | 0.284 | 1172_19561594 |
| magnitude_8 | 0.167 | 0.000 | 0.216 | 0.116 | 0.166 | 1173_19561594 |
| magnitude_9 | 0.130 | 0.000 | 0.383 | 0.270 | 0.327 | 1174_19561594 |
| magnitude_10 | 0.210 | 0.000 | 0.278 | 0.038 | 0.158 | 1175_19561594 |
| magnitude_11 | 0.084 | 0.010 | 0.438 | 0.210 | 0.324 | 1177_19561594 |
| magnitude_12 | 0.078 | 0.020 | 0.175 | 0.221 | 0.198 | 1213_8021254 |
| magnitude_13 | 0.097 | 0.002 | 0.136 | 0.177 | 0.157 | 1215_16041388 |
| magnitude_14 | 0.231 | 0.000 | 0.069 | 0.180 | 0.125 | 1216_19457263 |
| magnitude_15 | 0.218 | 0.000 | 0.391 | 0.140 | 0.265 | 1285_19304800 |
| magnitude_16 | 0.274 | 0.000 | 0.320 | 0.205 | 0.263 | 147_19457263 |
| magnitude_17 | 0.189 | 0.000 | 0.375 | 0.128 | 0.251 | 149_16041388 |
| magnitude_18 | 0.213 | 0.000 | 0.334 | 0.104 | 0.219 | 221_12324455 |
| magnitude_19 | 0.053 | 0.228 | 0.352 | 0.059 | 0.206 | 23_7510636 |
| magnitude_20 | 0.126 | 0.000 | 0.066 | 0.128 | 0.097 | 242_7543047 |
| magnitude_21 | 0.185 | 0.000 | 0.270 | 0.170 | 0.220 | 243_7543047 |
| magnitude_22 | 0.177 | 0.000 | 0.339 | 0.166 | 0.253 | 244_7543047 |
| magnitude_23 | 0.121 | 0.000 | 0.290 | 0.103 | 0.196 | 24_7908267 |
| magnitude_24 | 0.187 | 0.000 | 0.268 | 0.167 | 0.218 | 350_8846295 |
| magnitude_25 | 0.157 | 0.000 | 0.287 | 0.158 | 0.222 | 351_8846295 |
| magnitude_26 | 0.123 | 0.000 | 0.154 | 0.087 | 0.120 | 352_8846295 |
| magnitude_27 | 0.151 | 0.000 | 0.271 | 0.251 | 0.261 | 359_12507992 |
| magnitude_28 | 0.115 | 0.000 | 0.199 | 0.112 | 0.155 | 37_16537540 |
| magnitude_29 | 0.168 | 0.000 | 0.251 | 0.186 | 0.219 | 488_9001221 |
| magnitude_30 | 0.049 | 0.307 | 0.310 | 0.060 | 0.185 | 637_11098054 |
| magnitude_31 | 0.095 | 0.002 | 0.056 | 0.159 | 0.108 | 661_1717938 |
| magnitude_32 | 0.126 | 0.000 | 0.172 | 0.107 | 0.140 | 662_1717938 |
| magnitude_33 | 0.086 | 0.007 | 0.178 | 0.091 | 0.135 | 663_1717938 |
| magnitude_34 | 0.092 | 0.003 | 0.159 | 0.180 | 0.170 | 669_20071745 |
| magnitude_35 | 0.176 | 0.000 | 0.156 | 0.112 | 0.134 | 680_9789075 |
| magnitude_36 | 0.146 | 0.000 | 0.238 | 0.075 | 0.156 | 682_10811881 |
| magnitude_37 | 0.237 | 0.000 | 0.222 | 0.207 | 0.215 | 782_8497264 |
| magnitude_38 | 0.099 | 0.001 | 0.318 | 0.096 | 0.207 | 783_7972035 |
| magnitude_39 | 0.061 | 0.115 | 0.075 | 0.218 | 0.147 | 784_7972035 |
| magnitude_40 | 0.178 | 0.000 | 0.044 | 0.136 | 0.090 | 790_10094314 |
| magnitude_41 | 0.158 | 0.000 | 0.298 | 0.162 | 0.230 | 791_10094314 |
| magnitude_42 | 0.074 | 0.031 | 0.254 | 0.151 | 0.202 | 797_17548433 |
| magnitude_43 | 0.131 | 0.000 | 0.099 | 0.124 | 0.111 | 922_19282290 |
| magnitude_44 | 0.065 | 0.077 | 0.270 | 0.191 | 0.230 | 946_10094314 |
| magnitude_45 | 0.144 | 0.000 | 0.070 | 0.104 | 0.087 | 947_10094314 |
| magnitude_46 | 0.151 | 0.000 | 0.260 | 0.090 | 0.175 | 948_10094314 |
| magnitude_47 | 0.166 | 0.000 | 0.243 | 0.182 | 0.213 | 949_10094314 |
| magnitude_48 | 0.121 | 0.000 | 0.203 | 0.102 | 0.152 | 950_7908267 |
| magnitude_49 | 0.226 | 0.000 | 0.128 | 0.124 | 0.126 | 951_12324455 |
| magnitude_50 | 0.176 | 0.000 | 0.328 | 0.146 | 0.237 | 952_7543047 |
| magnitude_51 | 0.090 | 0.004 | 0.342 | 0.178 | 0.260 | 953_7543047 |
| magnitude_52 | 0.094 | 0.002 | 0.173 | 0.177 | 0.175 | 954_10094314 |
| magnitude_53 | 0.161 | 0.000 | 0.193 | 0.119 | 0.156 | 969_20167602 |
| magnitude_54 | 0.085 | 0.008 | 0.210 | 0.132 | 0.171 | SRSF_1.pssm |
| magnitude_55 | 0.193 | 0.000 | 0.121 | 0.117 | 0.119 | SRSF_2.pssm |
| magnitude_56 | 0.037 | 0.658 | 0.245 | 0.064 | 0.155 | SRSF_5.pssm |
| magnitude_57 | 0.087 | 0.006 | 0.095 | 0.131 | 0.113 | SRSF_6.pssm |
| magnitude_58 | 0.179 | 0.000 | 0.168 | 0.147 | 0.158 | M001_0.6 |
| magnitude_59 | 0.086 | 0.008 | 0.348 | 0.089 | 0.219 | M002_0.6 |
| magnitude_60 | 0.177 | 0.000 | 0.151 | 0.158 | 0.155 | M004_0.6 |
| magnitude_61 | 0.093 | 0.003 | 0.302 | 0.090 | 0.196 | M008_0.6 |
| magnitude_62 | 0.234 | 0.000 | 0.137 | 0.036 | 0.087 | M012_0.6 |
| magnitude_63 | 0.121 | 0.000 | 0.294 | 0.104 | 0.199 | M013_0.6 |
| magnitude_64 | 0.131 | 0.000 | 0.226 | 0.185 | 0.206 | M016_0.6 |
| magnitude_65 | 0.183 | 0.000 | 0.236 | 0.047 | 0.141 | M017_0.6 |
| magnitude_66 | 0.129 | 0.000 | 0.302 | 0.084 | 0.193 | M019_0.6 |
| magnitude_67 | 0.116 | 0.000 | 0.222 | 0.057 | 0.139 | M020_0.6 |
| magnitude_68 | 0.099 | 0.001 | 0.191 | 0.038 | 0.114 | M021_0.6 |
| magnitude_69 | 0.087 | 0.006 | 0.123 | 0.145 | 0.134 | M022_0.6 |
| magnitude_70 | 0.137 | 0.000 | 0.138 | 0.209 | 0.174 | M023_0.6 |
| magnitude_71 | 0.215 | 0.000 | 0.177 | 0.205 | 0.191 | M024_0.6 |
| magnitude_72 | 0.233 | 0.000 | 0.325 | 0.078 | 0.201 | M025_0.6 |
| magnitude_73 | 0.151 | 0.000 | 0.334 | 0.217 | 0.275 | M026_0.6 |
| magnitude_74 | 0.211 | 0.000 | 0.362 | 0.272 | 0.317 | M027_0.6 |
| magnitude_75 | 0.136 | 0.000 | 0.199 | 0.060 | 0.130 | M031_0.6 |
| magnitude_76 | 0.193 | 0.000 | 0.249 | 0.114 | 0.182 | M032_0.6 |
| magnitude_77 | 0.109 | 0.000 | 0.207 | 0.223 | 0.215 | M033_0.6 |
| magnitude_78 | 0.262 | 0.000 | 0.244 | 0.214 | 0.229 | M035_0.6 |
| magnitude_79 | 0.086 | 0.007 | 0.443 | 0.156 | 0.300 | M036_0.6 |
| magnitude_80 | 0.077 | 0.021 | 0.186 | 0.225 | 0.206 | M037_0.6 |
| magnitude_81 | 0.115 | 0.000 | 0.185 | 0.080 | 0.133 | M040_0.6 |
| magnitude_82 | 0.163 | 0.000 | 0.033 | 0.131 | 0.082 | M042_0.6 |
| magnitude_83 | 0.161 | 0.000 | 0.177 | 0.106 | 0.141 | M043_0.6 |
| magnitude_84 | 0.129 | 0.000 | 0.204 | 0.094 | 0.149 | M044_0.6 |
| magnitude_85 | 0.112 | 0.000 | 0.277 | 0.192 | 0.234 | M045_0.6 |
| magnitude_86 | 0.188 | 0.000 | 0.286 | 0.234 | 0.260 | M046_0.6 |
| magnitude_87 | 0.140 | 0.000 | 0.235 | 0.129 | 0.182 | M047_0.6 |
| magnitude_88 | 0.317 | 0.000 | 0.210 | 0.029 | 0.120 | M048_0.6 |
| magnitude_89 | 0.157 | 0.000 | 0.491 | 0.145 | 0.318 | M049_0.6 |
| magnitude_90 | 0.079 | 0.017 | 0.154 | 0.110 | 0.132 | M050_0.6 |
| magnitude_91 | 0.155 | 0.000 | 0.072 | 0.077 | 0.074 | M051_0.6 |
| magnitude_92 | 0.109 | 0.000 | 0.192 | 0.171 | 0.182 | M052_0.6 |
| magnitude_93 | 0.113 | 0.000 | 0.071 | 0.068 | 0.070 | M053_0.6 |
| magnitude_94 | 0.119 | 0.000 | 0.047 | 0.205 | 0.126 | M054_0.6 |
| magnitude_95 | 0.133 | 0.000 | 0.240 | 0.204 | 0.222 | M055_0.6 |
| magnitude_96 | 0.092 | 0.003 | 0.332 | 0.039 | 0.185 | M056_0.6 |
| magnitude_97 | 0.135 | 0.000 | 0.030 | 0.262 | 0.146 | M060_0.6 |
| magnitude_98 | 0.111 | 0.000 | 0.364 | 0.241 | 0.302 | M061_0.6 |
| magnitude_99 | 0.092 | 0.003 | 0.217 | 0.189 | 0.203 | M062_0.6 |
| magnitude_100 | 0.126 | 0.000 | 0.099 | 0.150 | 0.125 | M065_0.6 |
| magnitude_101 | 0.068 | 0.061 | 0.262 | 0.060 | 0.161 | M068_0.6 |
| magnitude_102 | 0.224 | 0.000 | 0.162 | 0.107 | 0.134 | M069_0.6 |
| magnitude_103 | 0.079 | 0.016 | 0.298 | 0.098 | 0.198 | M070_0.6 |
| magnitude_104 | 0.082 | 0.012 | 0.289 | 0.215 | 0.252 | M071_0.6 |
| magnitude_105 | 0.170 | 0.000 | 0.144 | 0.088 | 0.116 | M072_0.6 |
| magnitude_106 | 0.165 | 0.000 | 0.284 | 0.169 | 0.227 | M073_0.6 |
| magnitude_107 | 0.079 | 0.017 | 0.274 | 0.145 | 0.210 | M074_0.6 |
| magnitude_108 | 0.174 | 0.000 | 0.179 | 0.151 | 0.165 | M075_0.6 |
| magnitude_109 | 0.129 | 0.000 | 0.185 | 0.137 | 0.161 | M077_0.6 |
| magnitude_110 | 0.087 | 0.006 | 0.086 | 0.032 | 0.059 | M078_0.6 |
| magnitude_111 | 0.195 | 0.000 | 0.133 | 0.125 | 0.129 | M079_0.6 |
| magnitude_112 | 0.197 | 0.000 | 0.236 | 0.079 | 0.158 | M081_0.6 |
| magnitude_113 | 0.108 | 0.000 | 0.076 | 0.211 | 0.144 | M082_0.6 |
| magnitude_114 | 0.082 | 0.012 | 0.118 | 0.149 | 0.133 | M083_0.6 |
| magnitude_115 | 0.088 | 0.005 | 0.099 | 0.164 | 0.131 | M085_0.6 |
| magnitude_116 | 0.112 | 0.000 | 0.116 | 0.121 | 0.119 | M086_0.6 |
| magnitude_117 | 0.186 | 0.000 | 0.266 | 0.135 | 0.200 | M087_0.6 |
| magnitude_118 | 0.064 | 0.090 | 0.223 | 0.136 | 0.180 | M088_0.6 |
| magnitude_119 | 0.140 | 0.000 | 0.086 | 0.157 | 0.121 | M089_0.6 |
| magnitude_120 | 0.092 | 0.003 | 0.305 | 0.157 | 0.231 | M097_0.6 |
| magnitude_121 | 0.189 | 0.000 | 0.163 | 0.169 | 0.166 | M098_0.6 |
| magnitude_122 | 0.151 | 0.000 | 0.251 | 0.179 | 0.215 | M099_0.6 |
| magnitude_123 | 0.132 | 0.000 | 0.276 | 0.117 | 0.197 | M100_0.6 |
| magnitude_124 | 0.129 | 0.000 | 0.102 | 0.158 | 0.130 | M101_0.6 |
| magnitude_125 | 0.270 | 0.000 | 0.064 | 0.088 | 0.076 | M102_0.6 |
| magnitude_126 | 0.166 | 0.000 | 0.482 | 0.265 | 0.374 | M103_0.6 |
| magnitude_127 | 0.145 | 0.000 | 0.231 | 0.264 | 0.248 | M104_0.6 |
| magnitude_128 | 0.185 | 0.000 | 0.257 | 0.115 | 0.186 | M105_0.6 |
| magnitude_129 | 0.095 | 0.002 | 0.311 | 0.167 | 0.239 | M106_0.6 |
| magnitude_130 | 0.169 | 0.000 | 0.059 | 0.096 | 0.078 | M108_0.6 |
| magnitude_131 | 0.098 | 0.001 | 0.311 | 0.195 | 0.253 | M109_0.6 |
| magnitude_132 | 0.175 | 0.000 | 0.170 | 0.116 | 0.143 | M111_0.6 |
| magnitude_133 | 0.059 | 0.146 | 0.343 | 0.143 | 0.243 | M112_0.6 |
| magnitude_134 | 0.105 | 0.000 | 0.083 | 0.166 | 0.124 | M113_0.6 |
| magnitude_135 | 0.082 | 0.012 | 0.074 | 0.089 | 0.082 | M117_0.6 |
| magnitude_136 | 0.160 | 0.000 | 0.058 | 0.164 | 0.111 | M118_0.6 |
| magnitude_137 | 0.168 | 0.000 | 0.301 | 0.089 | 0.195 | M120_0.6 |
| magnitude_138 | 0.111 | 0.000 | 0.223 | 0.091 | 0.157 | M121_0.6 |
| magnitude_139 | 0.285 | 0.000 | 0.115 | 0.047 | 0.081 | M122_0.6 |
| magnitude_140 | 0.115 | 0.000 | 0.367 | 0.164 | 0.266 | M124_0.6 |
| magnitude_141 | 0.108 | 0.000 | 0.169 | 0.131 | 0.150 | M126_0.6 |
| magnitude_142 | 0.240 | 0.000 | 0.161 | 0.084 | 0.123 | M127_0.6 |
| magnitude_143 | 0.171 | 0.000 | 0.340 | 0.113 | 0.227 | M134_0.6 |
| magnitude_144 | 0.338 | 0.000 | 0.310 | 0.108 | 0.209 | M136_0.6 |
| magnitude_145 | 0.343 | 0.000 | 0.609 | 0.360 | 0.484 | M138_0.6 |
| magnitude_146 | 0.145 | 0.000 | 0.490 | 0.085 | 0.288 | M140_0.6 |
| magnitude_147 | 0.077 | 0.023 | 0.225 | 0.218 | 0.222 | M141_0.6 |
| magnitude_148 | 0.273 | 0.000 | 0.044 | 0.087 | 0.066 | M142_0.6 |
| magnitude_149 | 0.232 | 0.000 | 0.307 | 0.155 | 0.231 | M143_0.6 |
| magnitude_150 | 0.166 | 0.000 | 0.260 | 0.236 | 0.248 | M144_0.6 |
| magnitude_151 | 0.171 | 0.000 | 0.146 | 0.243 | 0.195 | M145_0.6 |
| magnitude_152 | 0.174 | 0.000 | 0.176 | 0.160 | 0.168 | M146_0.6 |
| magnitude_153 | 0.116 | 0.000 | 0.231 | 0.029 | 0.130 | M147_0.6 |
| magnitude_154 | 0.295 | 0.000 | 0.274 | 0.134 | 0.204 | M148_0.6 |
| magnitude_155 | 0.084 | 0.009 | 0.442 | 0.087 | 0.264 | M149_0.6 |
| magnitude_156 | 0.150 | 0.000 | 0.101 | 0.170 | 0.135 | M150_0.6 |
| magnitude_157 | 0.202 | 0.000 | 0.294 | 0.219 | 0.257 | M151_0.6 |
| magnitude_158 | 0.120 | 0.000 | 0.278 | 0.103 | 0.190 | M152_0.6 |
| magnitude_159 | 0.131 | 0.000 | 0.046 | 0.081 | 0.063 | M153_0.6 |
| magnitude_160 | 0.049 | 0.309 | 0.159 | 0.162 | 0.160 | M154_0.6 |
| magnitude_161 | 0.293 | 0.000 | 0.005 | 0.050 | 0.028 | M155_0.6 |
| magnitude_162 | 0.282 | 0.000 | 0.381 | 0.134 | 0.258 | M156_0.6 |
| magnitude_163 | 0.149 | 0.000 | 0.443 | 0.117 | 0.280 | M157_0.6 |
| magnitude_164 | 0.128 | 0.000 | 0.161 | 0.087 | 0.124 | M158_0.6 |
| magnitude_165 | 0.204 | 0.000 | 0.322 | 0.271 | 0.296 | M159_0.6 |
| magnitude_166 | 0.179 | 0.000 | 0.323 | 0.160 | 0.242 | M160_0.6 |
| magnitude_167 | 0.210 | 0.000 | 0.245 | 0.127 | 0.186 | M161_0.6 |
| magnitude_168 | 0.271 | 0.000 | 0.296 | 0.150 | 0.223 | M162_0.6 |
| magnitude_169 | 0.059 | 0.144 | 0.504 | 0.237 | 0.370 | M163_0.6 |
| magnitude_170 | 0.139 | 0.000 | 0.026 | 0.081 | 0.054 | M164_0.6 |
| magnitude_171 | 0.145 | 0.000 | 0.307 | 0.094 | 0.201 | M167_0.6 |
| magnitude_172 | 0.155 | 0.000 | 0.263 | 0.121 | 0.192 | M168_0.6 |
| magnitude_173 | 0.066 | 0.074 | 0.219 | 0.157 | 0.188 | M169_0.6 |
| magnitude_174 | 0.142 | 0.000 | 0.190 | 0.177 | 0.184 | M170_0.6 |
| magnitude_175 | 0.161 | 0.000 | 0.218 | 0.090 | 0.154 | M175_0.6 |
| magnitude_176 | 0.195 | 0.000 | 0.152 | 0.160 | 0.156 | M176_0.6 |
| magnitude_177 | 0.265 | 0.000 | 0.231 | 0.121 | 0.176 | M177_0.6 |
| magnitude_178 | 0.256 | 0.000 | 0.386 | 0.123 | 0.255 | M178_0.6 |
| magnitude_179 | 0.170 | 0.000 | 0.397 | 0.061 | 0.229 | M188_0.6 |
| magnitude_180 | 0.099 | 0.001 | 0.213 | 0.093 | 0.153 | M195_0.6 |
| magnitude_181 | 0.236 | 0.000 | 0.152 | 0.238 | 0.195 | M201_0.6 |
| magnitude_182 | 0.091 | 0.003 | 0.327 | 0.146 | 0.237 | M205_0.6 |
| magnitude_183 | 0.190 | 0.000 | 0.125 | 0.106 | 0.115 | M207_0.6 |
| magnitude_184 | 0.157 | 0.000 | 0.258 | 0.070 | 0.164 | M209_0.6 |
| magnitude_185 | 0.165 | 0.000 | 0.219 | 0.154 | 0.186 | M210_0.6 |
| magnitude_186 | 0.157 | 0.000 | 0.220 | 0.200 | 0.210 | M211_0.6 |
| magnitude_187 | 0.081 | 0.013 | 0.206 | 0.169 | 0.188 | M227_0.6 |
| magnitude_188 | 0.293 | 0.000 | 0.203 | 0.138 | 0.171 | M228_0.6 |
| magnitude_189 | 0.130 | 0.000 | 0.387 | 0.090 | 0.238 | M229_0.6 |
| magnitude_190 | 0.160 | 0.000 | 0.199 | 0.132 | 0.166 | M231_0.6 |
| magnitude_191 | 0.188 | 0.000 | 0.330 | 0.147 | 0.238 | M232_0.6 |
| magnitude_192 | 0.068 | 0.056 | 0.126 | 0.262 | 0.194 | M233_0.6 |
| magnitude_193 | 0.117 | 0.000 | 0.091 | 0.163 | 0.127 | M234_0.6 |
| magnitude_194 | 0.169 | 0.000 | 0.242 | 0.164 | 0.203 | M235_0.6 |
| magnitude_195 | 0.142 | 0.000 | 0.278 | 0.084 | 0.181 | M236_0.6 |
| magnitude_196 | 0.108 | 0.000 | 0.258 | 0.169 | 0.214 | M237_0.6 |
| magnitude_197 | 0.148 | 0.000 | 0.199 | 0.203 | 0.201 | M238_0.6 |
| magnitude_198 | 0.138 | 0.000 | 0.320 | 0.145 | 0.233 | M240_0.6 |
| magnitude_199 | 0.139 | 0.000 | 0.224 | 0.165 | 0.195 | M242_0.6 |
| magnitude_200 | 0.154 | 0.000 | 0.302 | 0.122 | 0.212 | M243_0.6 |
| magnitude_201 | 0.112 | 0.000 | 0.110 | 0.185 | 0.148 | M244_0.6 |
| post_prob_1 | 0.042 | 0.516 | 0.038 | 0.070 | 0.054 | 1004_8676391 |
| post_prob_2 | 0.106 | 0.000 | 0.018 | 0.070 | 0.044 | 1052_17318228 |
| post_prob_3 | 0.065 | 0.076 | 0.108 | 0.172 | 0.140 | 1053_17318228 |
| post_prob_4 | 0.102 | 0.001 | 0.117 | 0.043 | 0.080 | 1169_19561594 |
| post_prob_5 | 0.269 | 0.000 | 0.066 | 0.084 | 0.075 | 1170_19561594 |
| post_prob_6 | 0.039 | 0.597 | 0.348 | 0.199 | 0.273 | 1171_19561594 |
| post_prob_7 | 0.044 | 0.463 | 0.079 | 0.107 | 0.093 | 1172_19561594 |
| post_prob_8 | 0.014 | 1.000 | 0.061 | 0.085 | 0.073 | 1173_19561594 |
| post_prob_9 | 0.082 | 0.011 | 0.071 | 0.081 | 0.076 | 1174_19561594 |
| post_prob_10 | 0.250 | 0.000 | 0.066 | 0.060 | 0.063 | 1175_19561594 |
| post_prob_11 | 0.041 | 0.552 | 0.158 | 0.322 | 0.240 | 1177_19561594 |
| post_prob_12 | 0.054 | 0.216 | 0.071 | 0.047 | 0.059 | 1213_8021254 |
| post_prob_13 | 0.017 | 1.000 | 0.061 | 0.037 | 0.049 | 1215_16041388 |
| post_prob_14 | 0.235 | 0.000 | 0.047 | 0.000 | 0.023 | 1216_19457263 |
| post_prob_15 | 0.057 | 0.173 | 0.284 | 0.276 | 0.280 | 1285_19304800 |
| post_prob_16 | 0.025 | 0.973 | 0.011 | 0.078 | 0.045 | 147_19457263 |
| post_prob_17 | 0.033 | 0.789 | 0.000 | 0.086 | 0.043 | 149_16041388 |
| post_prob_18 | 0.107 | 0.000 | 0.071 | 0.000 | 0.035 | 221_12324455 |
| post_prob_19 | 0.061 | 0.119 | 0.118 | 0.209 | 0.163 | 23_7510636 |
| post_prob_20 | 0.050 | 0.293 | 0.035 | 0.070 | 0.053 | 242_7543047 |
| post_prob_21 | 0.045 | 0.433 | 0.007 | 0.177 | 0.092 | 243_7543047 |
| post_prob_22 | 0.069 | 0.055 | 0.059 | 0.134 | 0.096 | 244_7543047 |
| post_prob_23 | 0.027 | 0.951 | 0.038 | 0.117 | 0.077 | 24_7908267 |
| post_prob_24 | 0.209 | 0.000 | 0.051 | 0.091 | 0.071 | 350_8846295 |
| post_prob_25 | 0.145 | 0.000 | 0.284 | 0.194 | 0.239 | 351_8846295 |
| post_prob_26 | 0.018 | 1.000 | 0.107 | 0.141 | 0.124 | 352_8846295 |
| post_prob_27 | 0.098 | 0.001 | 0.033 | 0.088 | 0.061 | 359_12507992 |
| post_prob_28 | 0.028 | 0.933 | 0.074 | 0.028 | 0.051 | 37_16537540 |
| post_prob_29 | 0.205 | 0.000 | 0.041 | 0.019 | 0.030 | 488_9001221 |
| post_prob_30 | 0.062 | 0.103 | 0.197 | 0.231 | 0.214 | 637_11098054 |
| post_prob_31 | 0.040 | 0.584 | 0.002 | 0.080 | 0.041 | 661_1717938 |
| post_prob_32 | 0.039 | 0.619 | 0.050 | 0.076 | 0.063 | 662_1717938 |
| post_prob_33 | 0.023 | 0.986 | 0.005 | 0.043 | 0.024 | 663_1717938 |
| post_prob_34 | 0.056 | 0.180 | 0.022 | 0.038 | 0.030 | 669_20071745 |
| post_prob_35 | 0.077 | 0.023 | 0.056 | 0.085 | 0.070 | 680_9789075 |
| post_prob_36 | 0.022 | 0.993 | 0.105 | 0.068 | 0.087 | 682_10811881 |
| post_prob_37 | 0.272 | 0.000 | 0.004 | 0.043 | 0.023 | 782_8497264 |
| post_prob_38 | 0.098 | 0.001 | 0.327 | 0.209 | 0.268 | 783_7972035 |
| post_prob_39 | 0.070 | 0.045 | 0.128 | 0.109 | 0.118 | 784_7972035 |
| post_prob_40 | 0.050 | 0.305 | 0.003 | 0.042 | 0.023 | 790_10094314 |
| post_prob_41 | 0.159 | 0.000 | 0.011 | 0.135 | 0.073 | 791_10094314 |
| post_prob_42 | 0.032 | 0.836 | 0.255 | 0.047 | 0.151 | 797_17548433 |
| post_prob_43 | 0.100 | 0.001 | 0.047 | 0.063 | 0.055 | 922_19282290 |
| post_prob_44 | 0.044 | 0.440 | 0.090 | 0.169 | 0.129 | 946_10094314 |
| post_prob_45 | 0.048 | 0.345 | 0.070 | 0.026 | 0.048 | 947_10094314 |
| post_prob_46 | 0.124 | 0.000 | 0.026 | 0.043 | 0.034 | 948_10094314 |
| post_prob_47 | 0.096 | 0.002 | 0.170 | 0.084 | 0.127 | 949_10094314 |
| post_prob_48 | 0.129 | 0.000 | 0.080 | 0.131 | 0.105 | 950_7908267 |
| post_prob_49 | 0.074 | 0.030 | 0.116 | 0.124 | 0.120 | 951_12324455 |
| post_prob_50 | 0.095 | 0.002 | 0.013 | 0.023 | 0.018 | 952_7543047 |
| post_prob_51 | 0.061 | 0.122 | 0.028 | 0.194 | 0.111 | 953_7543047 |
| post_prob_52 | 0.025 | 0.967 | 0.098 | 0.087 | 0.092 | 954_10094314 |
| post_prob_53 | 0.018 | 1.000 | 0.058 | 0.095 | 0.077 | 969_20167602 |
| post_prob_54 | 0.015 | 1.000 | 0.055 | 0.106 | 0.080 | SRSF_1.pssm |
| post_prob_55 | 0.066 | 0.075 | 0.036 | 0.053 | 0.045 | SRSF_2.pssm |
| post_prob_56 | 0.066 | 0.071 | 0.097 | 0.075 | 0.086 | SRSF_5.pssm |
| post_prob_57 | 0.029 | 0.895 | 0.065 | 0.066 | 0.065 | SRSF_6.pssm |
| post_prob_58 | 0.164 | 0.000 | 0.002 | 0.106 | 0.054 | M001_0.6 |
| post_prob_59 | 0.115 | 0.000 | 0.095 | 0.174 | 0.135 | M002_0.6 |
| post_prob_60 | 0.087 | 0.006 | 0.185 | 0.073 | 0.129 | M004_0.6 |
| post_prob_61 | 0.000 | 1.000 | 0.007 | 0.107 | 0.057 | M008_0.6 |
| post_prob_62 | 0.096 | 0.002 | 0.000 | 0.000 | 0.000 | M012_0.6 |
| post_prob_63 | 0.092 | 0.003 | 0.078 | 0.097 | 0.087 | M013_0.6 |
| post_prob_64 | 0.109 | 0.000 | 0.029 | 0.222 | 0.126 | M016_0.6 |
| post_prob_65 | 0.130 | 0.000 | 0.042 | 0.142 | 0.092 | M017_0.6 |
| post_prob_66 | 0.042 | 0.523 | 0.047 | 0.258 | 0.153 | M019_0.6 |
| post_prob_67 | 0.101 | 0.001 | 0.043 | 0.160 | 0.102 | M020_0.6 |
| post_prob_68 | 0.061 | 0.112 | 0.151 | 0.128 | 0.140 | M021_0.6 |
| post_prob_69 | 0.057 | 0.163 | 0.026 | 0.083 | 0.054 | M022_0.6 |
| post_prob_70 | 0.029 | 0.905 | 0.022 | 0.172 | 0.097 | M023_0.6 |
| post_prob_71 | 0.044 | 0.445 | 0.063 | 0.052 | 0.057 | M024_0.6 |
| post_prob_72 | 0.040 | 0.566 | 0.027 | 0.036 | 0.031 | M025_0.6 |
| post_prob_73 | 0.125 | 0.000 | 0.046 | 0.100 | 0.073 | M026_0.6 |
| post_prob_74 | 0.144 | 0.000 | 0.032 | 0.180 | 0.106 | M027_0.6 |
| post_prob_75 | 0.050 | 0.301 | 0.157 | 0.150 | 0.153 | M031_0.6 |
| post_prob_76 | 0.040 | 0.579 | 0.075 | 0.109 | 0.092 | M032_0.6 |
| post_prob_77 | 0.071 | 0.042 | 0.033 | 0.000 | 0.016 | M033_0.6 |
| post_prob_78 | 0.123 | 0.000 | 0.065 | 0.134 | 0.100 | M035_0.6 |
| post_prob_79 | 0.284 | 0.000 | 0.112 | 0.208 | 0.160 | M036_0.6 |
| post_prob_80 | 0.038 | 0.641 | 0.157 | 0.318 | 0.238 | M037_0.6 |
| post_prob_81 | 0.085 | 0.008 | 0.036 | 0.140 | 0.088 | M040_0.6 |
| post_prob_82 | 0.078 | 0.019 | 0.095 | 0.129 | 0.112 | M042_0.6 |
| post_prob_83 | 0.088 | 0.005 | 0.071 | 0.171 | 0.121 | M043_0.6 |
| post_prob_84 | 0.063 | 0.095 | 0.141 | 0.107 | 0.124 | M044_0.6 |
| post_prob_85 | 0.047 | 0.362 | 0.141 | 0.245 | 0.193 | M045_0.6 |
| post_prob_86 | 0.096 | 0.002 | 0.076 | 0.085 | 0.080 | M046_0.6 |
| post_prob_87 | 0.156 | 0.000 | 0.138 | 0.026 | 0.082 | M047_0.6 |
| post_prob_88 | 0.199 | 0.000 | 0.164 | 0.076 | 0.120 | M048_0.6 |
| post_prob_89 | 0.053 | 0.236 | 0.136 | 0.232 | 0.184 | M049_0.6 |
| post_prob_90 | 0.119 | 0.000 | 0.095 | 0.069 | 0.082 | M050_0.6 |
| post_prob_91 | 0.098 | 0.001 | 0.086 | 0.147 | 0.117 | M051_0.6 |
| post_prob_92 | 0.039 | 0.619 | 0.129 | 0.077 | 0.103 | M052_0.6 |
| post_prob_93 | 0.141 | 0.000 | 0.065 | 0.075 | 0.070 | M053_0.6 |
| post_prob_94 | 0.044 | 0.441 | 0.190 | 0.134 | 0.162 | M054_0.6 |
| post_prob_95 | 0.091 | 0.004 | 0.089 | 0.094 | 0.092 | M055_0.6 |
| post_prob_96 | 0.041 | 0.528 | 0.023 | 0.143 | 0.083 | M056_0.6 |
| post_prob_97 | 0.258 | 0.000 | 0.044 | 0.019 | 0.032 | M060_0.6 |
| post_prob_98 | 0.017 | 1.000 | 0.124 | 0.344 | 0.234 | M061_0.6 |
| post_prob_99 | 0.085 | 0.008 | 0.040 | 0.158 | 0.099 | M062_0.6 |
| post_prob_100 | 0.088 | 0.005 | 0.068 | 0.089 | 0.078 | M065_0.6 |
| post_prob_101 | 0.117 | 0.000 | 0.109 | 0.061 | 0.085 | M068_0.6 |
| post_prob_102 | 0.087 | 0.006 | 0.112 | 0.026 | 0.069 | M069_0.6 |
| post_prob_103 | 0.243 | 0.000 | 0.000 | 0.096 | 0.048 | M070_0.6 |
| post_prob_104 | 0.039 | 0.602 | 0.171 | 0.355 | 0.263 | M071_0.6 |
| post_prob_105 | 0.061 | 0.116 | 0.087 | 0.026 | 0.056 | M072_0.6 |
| post_prob_106 | 0.132 | 0.000 | 0.064 | 0.123 | 0.093 | M073_0.6 |
| post_prob_107 | 0.086 | 0.007 | 0.053 | 0.114 | 0.083 | M074_0.6 |
| post_prob_108 | 0.075 | 0.028 | 0.056 | 0.103 | 0.079 | M075_0.6 |
| post_prob_109 | 0.046 | 0.383 | 0.099 | 0.166 | 0.132 | M077_0.6 |
| post_prob_110 | 0.020 | 0.997 | 0.104 | 0.049 | 0.076 | M078_0.6 |
| post_prob_111 | 0.049 | 0.328 | 0.046 | 0.072 | 0.059 | M079_0.6 |
| post_prob_112 | 0.257 | 0.000 | 0.102 | 0.061 | 0.081 | M081_0.6 |
| post_prob_113 | 0.128 | 0.000 | 0.286 | 0.246 | 0.266 | M082_0.6 |
| post_prob_114 | 0.033 | 0.809 | 0.189 | 0.191 | 0.190 | M083_0.6 |
| post_prob_115 | 0.155 | 0.000 | 0.057 | 0.075 | 0.066 | M085_0.6 |
| post_prob_116 | 0.027 | 0.944 | 0.115 | 0.144 | 0.129 | M086_0.6 |
| post_prob_117 | 0.046 | 0.389 | 0.019 | 0.000 | 0.010 | M087_0.6 |
| post_prob_118 | 0.040 | 0.567 | 0.067 | 0.140 | 0.103 | M088_0.6 |
| post_prob_119 | 0.043 | 0.483 | 0.000 | 0.047 | 0.024 | M089_0.6 |
| post_prob_120 | 0.044 | 0.448 | 0.057 | 0.136 | 0.097 | M097_0.6 |
| post_prob_121 | 0.016 | 1.000 | 0.012 | 0.130 | 0.071 | M098_0.6 |
| post_prob_122 | 0.051 | 0.277 | 0.000 | 0.037 | 0.019 | M099_0.6 |
| post_prob_123 | 0.157 | 0.000 | 0.075 | 0.115 | 0.095 | M100_0.6 |
| post_prob_124 | 0.023 | 0.985 | 0.068 | 0.222 | 0.145 | M101_0.6 |
| post_prob_125 | 0.224 | 0.000 | 0.073 | 0.049 | 0.061 | M102_0.6 |
| post_prob_126 | 0.179 | 0.000 | 0.058 | 0.294 | 0.176 | M103_0.6 |
| post_prob_127 | 0.074 | 0.030 | 0.238 | 0.159 | 0.199 | M104_0.6 |
| post_prob_128 | 0.046 | 0.392 | 0.112 | 0.150 | 0.131 | M105_0.6 |
| post_prob_129 | 0.028 | 0.924 | 0.000 | 0.173 | 0.087 | M106_0.6 |
| post_prob_130 | 0.024 | 0.984 | 0.087 | 0.095 | 0.091 | M108_0.6 |
| post_prob_131 | 0.043 | 0.476 | 0.096 | 0.116 | 0.106 | M109_0.6 |
| post_prob_132 | 0.105 | 0.000 | 0.047 | 0.071 | 0.059 | M111_0.6 |
| post_prob_133 | 0.052 | 0.247 | 0.009 | 0.154 | 0.081 | M112_0.6 |
| post_prob_134 | 0.055 | 0.200 | 0.066 | 0.116 | 0.091 | M113_0.6 |
| post_prob_135 | 0.097 | 0.002 | 0.023 | 0.055 | 0.039 | M117_0.6 |
| post_prob_136 | 0.014 | 1.000 | 0.100 | 0.000 | 0.050 | M118_0.6 |
| post_prob_137 | 0.014 | 1.000 | 0.068 | 0.000 | 0.034 | M120_0.6 |
| post_prob_138 | 0.027 | 0.937 | 0.063 | 0.035 | 0.049 | M121_0.6 |
| post_prob_139 | 0.149 | 0.000 | 0.054 | 0.048 | 0.051 | M122_0.6 |
| post_prob_140 | 0.044 | 0.460 | 0.197 | 0.076 | 0.137 | M124_0.6 |
| post_prob_141 | 0.096 | 0.002 | 0.050 | 0.000 | 0.025 | M126_0.6 |
| post_prob_142 | 0.062 | 0.111 | 0.061 | 0.046 | 0.054 | M127_0.6 |
| post_prob_143 | 0.061 | 0.116 | 0.064 | 0.060 | 0.062 | M134_0.6 |
| post_prob_144 | 0.401 | 0.000 | 0.065 | 0.020 | 0.042 | M136_0.6 |
| post_prob_145 | 0.086 | 0.007 | 0.452 | 0.410 | 0.431 | M138_0.6 |
| post_prob_146 | 0.017 | 1.000 | 0.023 | 0.105 | 0.064 | M140_0.6 |
| post_prob_147 | 0.077 | 0.022 | 0.013 | 0.043 | 0.028 | M141_0.6 |
| post_prob_148 | 0.025 | 0.969 | 0.065 | 0.066 | 0.065 | M142_0.6 |
| post_prob_149 | 0.034 | 0.774 | 0.114 | 0.099 | 0.107 | M143_0.6 |
| post_prob_150 | 0.088 | 0.005 | 0.000 | 0.000 | 0.000 | M144_0.6 |
| post_prob_151 | 0.122 | 0.000 | 0.098 | 0.149 | 0.124 | M145_0.6 |
| post_prob_152 | 0.055 | 0.197 | 0.010 | 0.292 | 0.151 | M146_0.6 |
| post_prob_153 | 0.021 | 0.996 | 0.054 | 0.118 | 0.086 | M147_0.6 |
| post_prob_154 | 0.025 | 0.974 | 0.003 | 0.034 | 0.018 | M148_0.6 |
| post_prob_155 | 0.041 | 0.550 | 0.000 | 0.017 | 0.009 | M149_0.6 |
| post_prob_156 | 0.114 | 0.000 | 0.077 | 0.052 | 0.065 | M150_0.6 |
| post_prob_157 | 0.065 | 0.081 | 0.025 | 0.241 | 0.133 | M151_0.6 |
| post_prob_158 | 0.097 | 0.002 | 0.036 | 0.110 | 0.073 | M152_0.6 |
| post_prob_159 | 0.111 | 0.000 | 0.088 | 0.140 | 0.114 | M153_0.6 |
| post_prob_160 | 0.052 | 0.260 | 0.056 | 0.097 | 0.076 | M154_0.6 |
| post_prob_161 | 0.056 | 0.176 | 0.044 | 0.067 | 0.056 | M155_0.6 |
| post_prob_162 | 0.157 | 0.000 | 0.000 | 0.064 | 0.032 | M156_0.6 |
| post_prob_163 | 0.171 | 0.000 | 0.172 | 0.176 | 0.174 | M157_0.6 |
| post_prob_164 | 0.049 | 0.325 | 0.131 | 0.135 | 0.133 | M158_0.6 |
| post_prob_165 | 0.075 | 0.028 | 0.055 | 0.130 | 0.093 | M159_0.6 |
| post_prob_166 | 0.107 | 0.000 | 0.001 | 0.172 | 0.086 | M160_0.6 |
| post_prob_167 | 0.043 | 0.494 | 0.153 | 0.005 | 0.079 | M161_0.6 |
| post_prob_168 | 0.123 | 0.000 | 0.069 | 0.100 | 0.084 | M162_0.6 |
| post_prob_169 | 0.066 | 0.075 | 0.022 | 0.237 | 0.130 | M163_0.6 |
| post_prob_170 | 0.134 | 0.000 | 0.016 | 0.086 | 0.051 | M164_0.6 |
| post_prob_171 | 0.071 | 0.044 | 0.026 | 0.160 | 0.093 | M167_0.6 |
| post_prob_172 | 0.062 | 0.107 | 0.058 | 0.000 | 0.029 | M168_0.6 |
| post_prob_173 | 0.042 | 0.498 | 0.107 | 0.053 | 0.080 | M169_0.6 |
| post_prob_174 | 0.112 | 0.000 | 0.066 | 0.087 | 0.076 | M170_0.6 |
| post_prob_175 | 0.166 | 0.000 | 0.108 | 0.123 | 0.115 | M175_0.6 |
| post_prob_176 | 0.143 | 0.000 | 0.052 | 0.128 | 0.090 | M176_0.6 |
| post_prob_177 | 0.019 | 0.999 | 0.097 | 0.194 | 0.145 | M177_0.6 |
| post_prob_178 | 0.161 | 0.000 | 0.008 | 0.078 | 0.043 | M178_0.6 |
| post_prob_179 | 0.123 | 0.000 | 0.247 | 0.186 | 0.216 | M188_0.6 |
| post_prob_180 | 0.015 | 1.000 | 0.065 | 0.091 | 0.078 | M195_0.6 |
| post_prob_181 | 0.033 | 0.813 | 0.000 | 0.006 | 0.003 | M201_0.6 |
| post_prob_182 | 0.109 | 0.000 | 0.086 | 0.000 | 0.043 | M205_0.6 |
| post_prob_183 | 0.062 | 0.107 | 0.036 | 0.131 | 0.083 | M207_0.6 |
| post_prob_184 | 0.062 | 0.110 | 0.097 | 0.039 | 0.068 | M209_0.6 |
| post_prob_185 | 0.020 | 0.997 | 0.110 | 0.049 | 0.079 | M210_0.6 |
| post_prob_186 | 0.076 | 0.025 | 0.034 | 0.146 | 0.090 | M211_0.6 |
| post_prob_187 | 0.045 | 0.435 | 0.061 | 0.124 | 0.092 | M227_0.6 |
| post_prob_188 | 0.154 | 0.000 | 0.005 | 0.159 | 0.082 | M228_0.6 |
| post_prob_189 | 0.094 | 0.002 | 0.144 | 0.177 | 0.161 | M229_0.6 |
| post_prob_190 | 0.021 | 0.995 | 0.000 | 0.073 | 0.037 | M231_0.6 |
| post_prob_191 | 0.054 | 0.219 | 0.035 | 0.104 | 0.069 | M232_0.6 |
| post_prob_192 | 0.034 | 0.757 | 0.042 | 0.154 | 0.098 | M233_0.6 |
| post_prob_193 | 0.052 | 0.246 | 0.018 | 0.107 | 0.062 | M234_0.6 |
| post_prob_194 | 0.046 | 0.390 | 0.020 | 0.048 | 0.034 | M235_0.6 |
| post_prob_195 | 0.043 | 0.490 | 0.005 | 0.124 | 0.064 | M236_0.6 |
| post_prob_196 | 0.281 | 0.000 | 0.056 | 0.093 | 0.074 | M237_0.6 |
| post_prob_197 | 0.133 | 0.000 | 0.182 | 0.270 | 0.226 | M238_0.6 |
| post_prob_198 | 0.025 | 0.967 | 0.068 | 0.184 | 0.126 | M240_0.6 |
| post_prob_199 | 0.017 | 1.000 | 0.045 | 0.000 | 0.023 | M242_0.6 |
| post_prob_200 | 0.012 | 1.000 | 0.090 | 0.000 | 0.045 | M243_0.6 |
| post_prob_201 | 0.076 | 0.024 | 0.037 | 0.099 | 0.068 | M244_0.6 |

*Description of each column: Feature: brief name of features used in our model; D value(K-S test): D value for K-S test performed on individual feature, between positive and negative datasets; P-value(K-S test):P value for the K-S test; MCC (on_ss): Matthew correlation coefficient for individual feature, for variants on splicing site; MCC(off_ss): Matthew correlation coefficient for individual feature, for variants off splicing site; average MCC: the average of MCC values for features used both in on-splicing site and off-splicing site model; Feature description: a brief description of one feature.
